# Genomic, transcriptomic, and metabolic characterization of 2-Phenylethanol-resistant *Saccharomyces cerevisiae* obtained by evolutionary engineering

**DOI:** 10.3389/fmicb.2023.1148065

**Published:** 2023-04-11

**Authors:** Can Holyavkin, Burcu Turanlı-Yıldız, Ülkü Yılmaz, Ceren Alkım, Mevlüt Arslan, Alican Topaloğlu, Halil İbrahim Kısakesen, Gustavo de Billerbeck, Jean Marie François, Z. Petek Çakar

**Affiliations:** ^1^Department of Molecular Biology & Genetics, Faculty of Science & Letters, Istanbul Technical University, Istanbul, Turkey; ^2^Dr. Orhan Öcalgiray Molecular Biology, Biotechnology and Genetics Research Center (ITU-MOBGAM), Istanbul Technical University, Istanbul, Turkey; ^3^INSA, UPS, INP, Université de Toulouse, Toulouse, France; ^4^Toulouse Biotechnology Institute (TBI), CNRS, INRA, INSA, Université de Toulouse, Toulouse, France

**Keywords:** adaptive laboratory evolution, environmental stress response, evolutionary engineering, genomic analysis, 2-phenylethanol, *Saccharomyces cerevisiae*, stress resistance, transcriptomic analysis

## Abstract

2-Phenylethanol is an aromatic compound commonly used in the food, cosmetic, and pharmaceutical industries. Due to increasing demand for natural products by consumers, the production of this flavor by microbial fermentation is gaining interest, as a sustainable alternative to chemical synthesis or expensive plant extraction, both processes relying on the use of fossil resources. However, the drawback of the fermentation process is the high toxicity of 2-phenylethanol to the producing microorganism. The aim of this study was to obtain a 2-phenylethanol-resistant *Saccharomyces cerevisiae* strain by *in vivo* evolutionary engineering and characterize the adapted yeast at the genomic, transcriptomic and metabolic levels. For this purpose, the tolerance to 2-phenylethanol was developed by gradually increasing the concentration of this flavor compound through successive batch cultivations, leading to an adapted strain that could tolerate 3.4 g/L of 2-phenylethanol, which was about 3-times better than the reference strain. Genome sequencing of the adapted strain identified point mutations in several genes, notably in *HOG1* that encodes the Mitogen-Activated Kinase of the high-osmolarity signaling pathway. As this mutation is localized in the phosphorylation lip of this protein, it likely resulted in a hyperactive protein kinase. Transcriptomic analysis of the adapted strain supported this suggestion by revealing a large set of upregulated stress-responsive genes that could be explained in great part by *HOG1*-dependent activation of the Msn2/Msn4 transcription factor. Another relevant mutation was found in *PDE2* encoding the low affinity cAMP phosphodiesterase, the missense mutation of which may lead to hyperactivation of this enzyme and thereby enhance the stressful state of the 2-phenylethanol adapted strain. In addition, the mutation in *CRH1* that encodes a chitin transglycosylase implicated in cell wall remodeling could account for the increased resistance of the adapted strain to the cell wall-degrading enzyme lyticase. Finally, the potent upregulation of *ALD3* and *ALD4* encoding NAD^+^ -dependent aldehyde dehydrogenase together with the observed phenylacetate resistance of the evolved strain suggest a resistance mechanism involving conversion of 2-phenylethanol into phenylacetaldehyde and phenylacetate implicating these dehydrogenases.

## 1. Introduction

2-Phenylethanol (2-PE) is an aromatic alcohol compound that is widely used in the cosmetic, perfume, and food industries. This flavor compound is mainly produced by chemical synthesis at a world production of about 10,000 tons per year with a market value of about 280 million USD (Mitri et al., 2022). The chemical synthesis involves three different processes which include the Friedel-Crafts reaction of ethylene oxide with benzene in the presence of aluminum chloride, the hydrogenation of styrene oxide with sodium hydroxide and Raney nickel as a catalyst, and the oxidation of propylene with 2-phenylethyl hydroperoxide. However, the use of toxic and carcinogenic chemicals such as benzene and styrene oxide is a major drawback. Additionally, as 2-PE is used in food and pharmaceuticals, safety regulations advocate the use of natural production methods, which are also decided by consumer plebiscites. These natural methods include solvent-based extraction mainly from rose flowers and biotechnological processes, either by bioconversion of phenylalanine or directly from glucose using different yeast species (Mitri et al., 2022).

The biotechnological production of 2-PE by yeasts is limited by its high toxicity, as full inhibition of growth has been observed in the range of 1.5 to 4.0 g/L, depending on the yeast (*Saccharomyces cerevisiae*) strain and species used (Carlquist et al., 2015). Concentrations above 4.0 g/L was shown to cause cell death (Vargai et al., 2018). Various potential targets have been proposed in the literature to account for the mechanism by which 2-PE exerts its toxicity. Among others, increasing membrane fluidization (Ingram and Buttke, 1984), ion leakage (Seward et al., 1996), and consequently, reduction of amino acids and glucose uptake (Lester, 1965) were the most frequently considered. It was also reported that 2-PE can induce respiration deficiency by increasing mitochondrial membrane permeability in the yeast *S. cerevisiae* (Wilkie and Maroudas, 1969). There are also some reports on the inhibitory effects of 2-PE on DNA synthesis (Bostock, 1970), RNA synthesis and glucose incorporation (Burns and Wang, 1968).

To increase the efficiency of microbial 2-PE production, microorganisms that can tolerate high levels of 2-PE need to be developed. For the improvement of phenotypes with an unknown molecular basis, evolutionary engineering, also known as adaptive laboratory evolution (ALE), is a highly promising approach (Mavrommati et al., 2022). It allows the selection of the desired phenotype by following the rules of natural evolution, without preliminary information. It starts with a random mutagenesis step that increases the starting population’s genetic diversity and continues with exposure to selective pressure for obtaining the desired phenotypes (Çakar et al., 2012). In this work, we used this *in vivo* evolutionary engineering strategy that enabled *S. cerevisiae* to adapt to 3.4 g/L 2-PE, where the 2-PE-tolerance of the evolved strain became about 3 times higher than the original strain. We then characterized this adapted strain at the genomic, metabolic, and transcriptomic levels. Overall, major findings of this work were that the evolved strain acquired a physiological state reflecting the typical environmental stress response (ESR). This stressful state of the 2-PE-adapted strain could be very likely the result of a mutation in the *HOG1* gene leading to a hyperactive MAPKinase that in turn activates the Msn2/4p transcription factor. In addition, the extraordinary transcriptional activation of *ALD3* gene encoding a NAD^+^ -dependent aldehyde dehydrogenase known to oxidize 2-phenylacetaldehyde into the less toxic 2-phenylacetate (2-PEA) may be the direct mechanism to reduce the toxicity effect of 2-PE in yeast.

## 2. Materials and methods

### 2.1. Strain, media, and growth conditions

The prototrophic haploid *Saccharomyces cerevisiae* CEN.PK 113.7D (MATa, MAL2-8c, SUC2) (Entian and Kötter, 2007) was used as the reference strain throughout the study. Ethyl methanesulfonate (EMS) mutagenesis was applied to the reference strain to generate a genetically diverse initial population, as described previously (Lawrence, 1991; Çakar et al., 2009). EMS mutagenesis was carried out to allow 10% of the initial population to survive.

Yeast peptone dextrose (YPD) medium containing 10 g/L yeast extract, 20 g/L dextrose, 10 g/L peptone; and yeast minimal medium (YMM) containing 20 g/L dextrose and 6.7 g/L yeast nitrogen base without amino acids were used for cultivation. Agar was added to a final concentration of 20 g/L when solid media were prepared. Cultivations were done at 30°C, 150 rpm in 50-mL culture tubes with 10 ml culture volume unless otherwise stated. Optical density measurements at 600 nm (OD_600_) were used to monitor growth by using a spectrophotometer (Shimadzu UV-1700). Cultures were stored at −80°C as 2 ml YMM culture aliquots containing 30% (v/v) glycerol.

### 2.2. Selection of 2-phenylethanol-resistant strains by evolutionary engineering

The EMS-mutagenized culture was used as the initial population for the selection procedure. As an evolutionary engineering strategy, successive batch selection was performed by gradually increasing the concentration of 2-phenylethanol (2-PE) from 1.5 up to 3.4 g/L, as described below. The initial stress level of 1.5 g/L was chosen based on minimum inhibitory concentration (MIC) determination for 2-PE for both reference strain and the EMS-mutagenized initial population.

For selection experiments, first, the EMS-mutagenized culture was inoculated into 10 ml of YMM that contained 1.5 g/L 2-PE. The culture was then incubated at 30°C, 150 rpm, for 24 h, centrifuged at 10,000 g for 5 min using a benchtop centrifuge (Eppendorf, Centrifuge 5424, Germany), and washed twice with fresh YMM. The culture was then inoculated into fresh YMM with a gradually increased 2-PE concentration at an initial OD_600_ of 0.3 (approximately 3×10^6^ cells/mL) in 10 ml culture volume. The first culture grown in the presence of 1.5 g/L 2-PE was named the first passage. The second passage was obtained by transferring the first passage to a fresh YMM containing 1.6 g/L 2-PE to an initial OD_600_ of 0.3. For each passage, the cultures were also incubated under non-stress (control) conditions (YMM only). These reference cultures were used to calculate the survival rates for each passage. At each passage, 2-PE concentrations were gradually increased by 0.1 g/L until 2.8 g/L (passage n^o^:14). At 2.8 g/L and higher 2-PE concentrations, the same 2-PE concentration was used for more than one passage before increasing the concentration by 0.1 g/L, until 3.4 g/L at the 56th passage, the final population of selection. The final population was plated on solid YMM upon serial dilution. Ten individual colonies were randomly picked from plates for further analyses.

### 2.3. Quantification of 2-PE tolerance and cross-resistances to various stresses

2-PE-resistance of individual mutants was quantitatively estimated using the high-throughput, most-probable number (MPN) method, as described previously (Russek and Colwell, 1983; Çakar et al., 2005). Briefly, serial dilutions in the range of 10^−1^ to 10^−8^ were made in 96-well plates that contained 180 μL YMM (control) and 3 g/L 2-PE in 180 μL YMM (stress condition), as five replicates. After 72 h of incubation at 30°C, viable cell numbers were estimated using MPN tables (Lindquist, 2016). 2-PE-resistance was expressed as “survival rate”, and it was calculated by dividing the number of viable cells that survived 3 g/L 2-PE with respect to the number of viable cells in the absence of 2-PE (Çakar et al., 2009; Küçükgöze et al., 2013).

**Figure 1:**
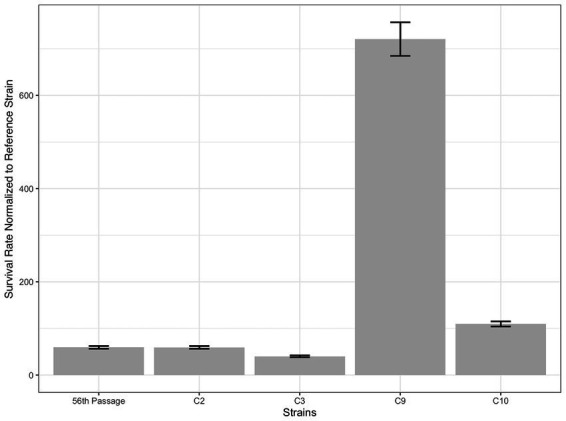
2-phenylethanol (2-PE) resistance and cross-resistance test results of the 2-PE-resistant evolved strain C9 and the reference strain (Ref) upon 2-PE (2.5 g/L and 3 g/L), boron (80 mM), cobalt (1 mM and 3 mM), NaCl (0.5 M), and phenylacetate (2 mM) stresses.

Resistance to 2-PE and other stress conditions were estimated using spot assay based on serial dilutions on solid culture media (Küçükgöze et al., 2013). Briefly, overnight precultures grown at 30°C in 10 mL YMM until their late logarithmic phase of growth were concentrated to 4 OD_600_ units by centrifugation at 10,000 g for 1 min and resuspended in 50 μL sterile YMM. They were then serially diluted between 10^−1^ to 10^−5^ and 5 μL of each dilution were spotted on YMM plates (control) and YMM plates containing various stress factors: potassium acetate, boron, cobalt, copper, nickel, ethanol, hydrogen peroxide, 2-PE, NaCl, sorbitol, phenylacetaldehyde, and phenylacetate. The stressors were added at concentrations indicated in Figure 1 and Supplementary Figure S2. Plates were incubated at 30°C for 72 h.

### 2.4. Whole-genome sequencing

For comparative whole-genome sequencing, the reference and evolved strain cultures were grown and collected as described previously (Sürmeli et al., 2019). For DNA extraction, a MasterPureTM DNA Purification Kit (Epicenter, San Diego, United States) was used, according to the manufacturer’s instructions. DNA concentration and purity was determined using NanoDrop 2000 UV–Vis spectrophotometer (Thermo Fisher Scientific, Waltham, MA, United States) and QubitR Fluorometer 3.0 (ThermoFisher Scientific). The genomic DNA libraries were prepared from 100 ng isolated DNA using Ion Xpress Plus Fragment Library Kit (ThermoFisher) and Ion 540™ Chip Kit according to the manufacturer’s protocol. The whole-genome sequencing was carried out on Ion S5 Next-Generation Sequencing Platform (ThermoFisher) and coupled automated library prep platform Ion ChefTM (ThermoFisher). Raw data were deposited in the NCBI Sequence Read Archive (SRA) database under BioProject PRJNA601278.

The quality of the raw data was evaluated by FastQC v.0.11.5 (Babraham Bioinformatics). Low-quality bases and remnant adapters were trimmed using Trimmomatic v.0.32 (Bolger et al., 2014). The reads of both reference and resistant strains were aligned to the previously assembled sequence of *S. cerevisiae* CEN.PK113-7D (GCA_000269885.1) (Nijkamp et al., 2012) using the Burrows-Wheeler aligner MEM v.0.7.1 (Li and Durbin, 2009). Mutations were called by using Genome Analysis Toolkit (GATK) v.3.8.0 (DePristo et al., 2011) and were manually inspected with GenomeBrowse v2.1.2 (GoldenHelix). The variants with low quality and common in both reference and the evolved strains were filtered out using in-house R scripts. Unique mutations of the evolved strain were annotated using Variant Effect Predictor v.90 using the latest gene build *S. cerevisiae* CEN.PK113-7D, ASM26988v1.

### 2.5. Whole-genome transcriptomic analysis

For comparative whole-genome transcriptomic analysis of the selected 2-PE-resistant evolved strain and the reference strain, Agilent yeast microarray systems were used, as described previously (Küçükgöze et al., 2013). Briefly, three replicates of each culture were grown in 100 ml YMM, using 500 ml flasks, at 30°C and 150 rpm. Cells at about 10^7^ cells/mL (~1 OD_600_) were used for total RNA isolation by RNeasy Mini Kit (Qiagen). RNA concentrations were determined by using a microvolume UV–Vis spectrophotometer (NanoDrop 2000). The RNA integrity numbers (RIN) were determined using Agilent Bioanalyzer 2100 and the relevant RNA 6000 Nano Assay Kit, according to the instructions of the manufacturer. RIN values of all RNA samples included in the transcriptomic analysis were higher than 8.

Internal controls were provided by Agilent One-Color RNA Spike-In Kit. cRNA samples were labeled with cyanine 3 (Cy3), and purified using Absolutely RNA Nanoprep Kit (Agilent). Hybridization of the samples on microarray slides was done at 65°C for 17 h, using Microarray Hybridization Chamber Kit (Agilent). Following washing steps, the microarray slides were scanned, and the data were analyzed by using Agilent Feature Extraction Software (Version 10.7) and GeneSpring GX Data Analysis Software (Version 12.0). Student’s *t*-test and Benjamini and Hochberg’s *p* value correction and false discovery rate (FDR) (Benjamini and Hochberg, 1995) were used were used for reliability assessment of gene expression data. For significantly different gene expression analysis, corrected *p* values <0.05 were considered. Additionally, genes with two-fold or higher expression changes (increase and decrease) and a *p* value of less than or equal to 0.01 were considered for cluster analysis. For all samples, the microarray experiment was performed in triplicate, with three independent cultures. FunSpec online software (Robinson et al., 2002) and FunCat database^1^ (Ruepp et al., 2004) were used for data classification into clusters and functional categories. To compare the gene expression data with Environmental Stress Response (ESR)-induced and ESR-repressed gene sets in yeast, “Yeast Growth Rate Homepage”^2^ (Brauer et al., 2008) was used. Pathway enrichment analyses were performed using the Limma package in R/Bioconductor (Ritchie et al., 2015) based on the Kyoto Encyclopedia of Genes and Genomes (KEGG) pathway database. This work is fully MIAME-compliant and the complete microarray data have been deposited at GEO repository. The accession number is GSE59353 at.^3^

### 2.6. Physiological analyses

The physiological properties of the evolved strain and the reference strain were determined by obtaining their growth and metabolite profiles. All physiological analyses were performed under both control (YMM) and stress (YMM + 3 g/L 2-PE) conditions, at 30°C and 150 rpm. Initially, both strains were incubated overnight in 10 ml YPD and then precultured overnight in 10 mL YMM for 24 h, using 50 mL-culture tubes. The cultures were inoculated into 50 mL control (YMM) and stress (YMM + 3 g/L 2-PE) medium in 250 mL-flasks to an initial OD_600_ of 0.2 (approximately 2×10^6^ cells/mL). One milli liter-samples were taken from both cultures at one-hour intervals. These samples were used in OD_600_ measurements for cell growth profiling and for metabolite measurements, in triplicates. For metabolite measurements, 1.5 mL-culture samples were centrifuged at 15,000 g for 5 min, and the supernatants were filtered using 0.22 μm filters. The filtered supernatants were used for metabolite measurements, including glucose, ethanol, glycerol and acetate, using HPLC (Shimadzu Series 10A HPLC, Shimadzu Co., Kyoto, Japan) at 60°C. As the mobile phase, 5 mM H_2_SO_4_ was used at a flow rate of 0.6 ml/min, as described previously (Küçükgöze et al., 2013). RID-10A refractive-index detector and an Aminex© HPX-87H ion exclusion column (300 mm × 7.8 mm, Bio-Rad Laboratories, CA, United States) were also used for the measurements. For final cell dry weight (CDW) analysis, 2 ml-culture samples were centrifuged in preweighed microfuge tubes, centrifuged at 15,000 g for 5 min, the supernatants were discarded and the pellets were dried at 80°C for 12 h. The tubes were then cooled in a desiccator for 90 min and reweighed.

Trehalose and glycogen contents were determined as described previously (Küçükgöze et al., 2013), based on an enzymatic method (Parrou and François, 1997).

Lyticase sensitivity assay was performed according to Kuranda et al. (2006) with slight modifications, as described previously (Kocaefe-Özşen et al., 2022). Briefly, overnight cultures of the evolved and the reference strain, grown both in the presence and absence (control) of 3 g/L 2-PE stress were harvested upon centrifugation at 10,000 g for 10 min. Cell pellets were dissolved in 10 ml of 10 mM Tris/HCl buffer (pH 7.4), including 40 mM β-mercaptoethanol. Upon incubation at 25°C for 30 min, 2 U/ml lyticase was added and the samples were incubated at 30°C and agitated at 150 rpm. OD_600_ measurements were taken every 20 min. Lyticase sensitivity was monitored by measuring the decrease in OD_600_, and lyticase resistance was calculated by dividing the measured OD_600_ values by the initial OD_600_ value and multiplying the result by 100. The experiments were performed as three biological repeats.

### 2.7. Statistical analysis

At least three biological repeats were made for all experiments. The R software “stats” package (R Core Team, 2020) was used for data analyses. To calculate statistical significance, a two-tailed, unpaired Student’s *t*-test was used, and *p* < 0.05 was considered as statistically significant, except for microarray and whole genome sequencing analyses.

## 3. Results

### 3.1. Experimental conditions for evolutionary engineering of 2-PE resistance

Before starting with the selection experiments, the 2-PE concentration that reduced 50% of the growth of both the reference strain and the EMS-mutagenized initial population was determined as 1.5 g/L (see Supplementary Table S1). Therefore, this concentration of 2-PE was chosen as the initial stress level for selection by evolutionary engineering. The evolutionary engineering experiments were performed with the EMS-mutagenized initial population of the prototrophic *S. cerevisiae* strain CEN.PK113-7D, as described in the Materials and Methods section. Briefly, successive batch selection was applied in YMM with gradually increased 2-PE concentrations starting from 1.5 g/L up to 3.4 g/L, for 56 passages (about 224 generations). As the survival rate significantly decreased to less than 60% at the 56th passage corresponding to 3.4 g/L 2-PE stress, the evolutionary selection experiment was stopped at that passage, named as the final population of selection. This final population was plated on solid YMM to pick 10 individual colonies randomly for further characterization.

### 3.2. Stress resistance characteristics of selected evolved strains

**Figure 2:**
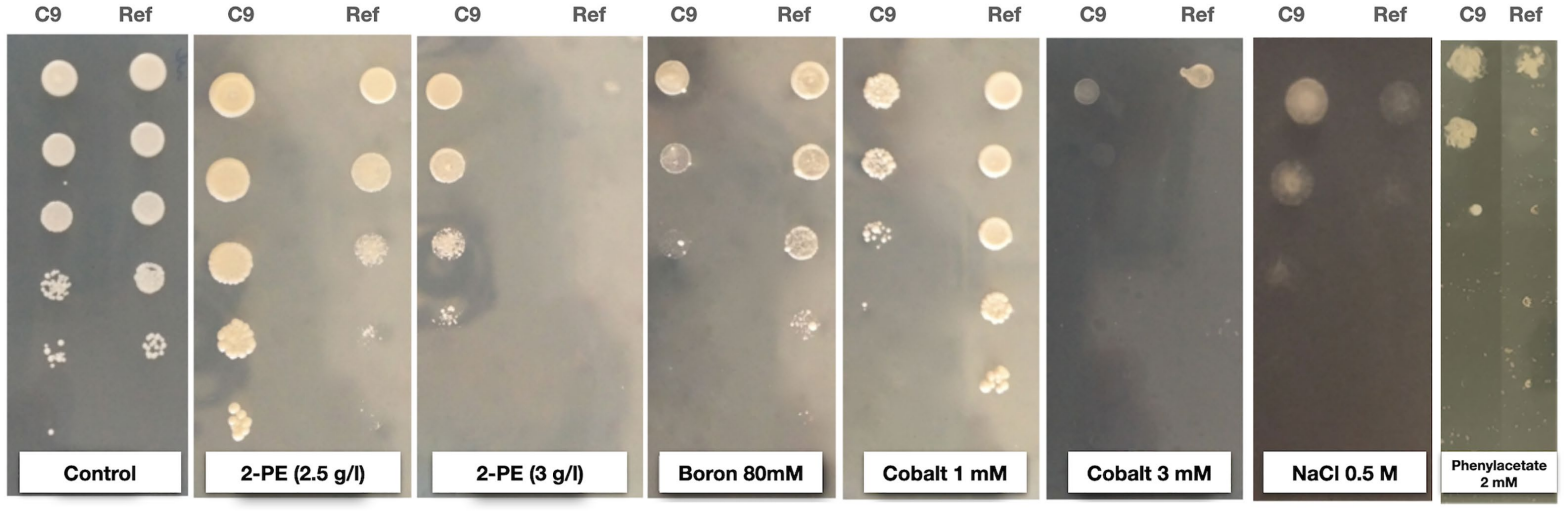
Survival rates of the final population of selection (56th passage), and the individual evolved strains C2, C3, C9, and C10, selected from the final population, in the presence of 3 g/L 2-phenylethanol stress. The survival rates are shown as fold of the reference strain’s survival rate.

The reference strain, the final population and the ten individual colonies (C1 to C10), were tested for their 2-PE resistance by spot assay on YMM plates. It was found that C2, C3, C5, C9, and C10 were able to grow in the presence of 3 g/L 2-PE (Supplementary Figure S1), while the reference strain could barely grow at this 2-PE concentration. At 5 g/L of 2-PE on the YMM plates, neither the final population nor the isolated clones were able to grow (data not shown). Additional quantitative estimation of 2-PE resistance of the reference strain, final population and individual clones C2, C3, C9 and C10 was done using the MPN method by growing them in microtiter plates in YMM containing 3 g/L 2-PE for 72 h at 30°C. As shown in Figure 2, the survival rate of the final selection population was approximately 59 times higher than that of the reference strain. It is also shown that the survival rate of the isolated clones was 40 to more than 700 times higher than that of the reference strain, which illustrates a large heterogeneity in the resistance of the final population to 2-PE and thus indicates that the acquisition of this resistance is largely pleiotropic. The evolved strain C9 that exhibited the highest survival rate was therefore retained and further analyzed for the genetic stability of this phenotypic trait by performing five successive batch growth cycles (about 20 generations) in a rich YPD medium in the absence of 2-PE stress, showing no loss of resistance levels after this successive cycle (data not shown).

When yeast cells gain resistance against a certain stress type, they may also become resistant against other stress types (Lewis et al., 1995; Hohmann, 2002). For this reason, we tested the 2-PE-resistant strain C9 and the reference strain (as control) for their potential cross-resistance against various stress types. Cross-resistance to other stressors was carried out by spot assay, revealing that the tolerance of C9 to cobalt and boron was slightly lower than that of the reference strain. In contrast, the C9 strain exhibited a slightly higher tolerance to NaCl and a significantly better resistance to 2- phenylacetate (Figure 1). The observed cross-resistance of the evolved strain against 2-phenylacetate may be associated with a 2-PE resistance mechanism that involves conversion of 2-phenylethanol into phenylacetaldehyde and phenylacetate. Interestingly, sensitivity to phenylacetaldehyde was similar for C9 and the reference as well as for ethanol, acetate, sorbitol, copper, nickel, and H_2_O_2_ (Supplementary Figure S2).

### 3.3. Physiological analyses

**Figure 3:**
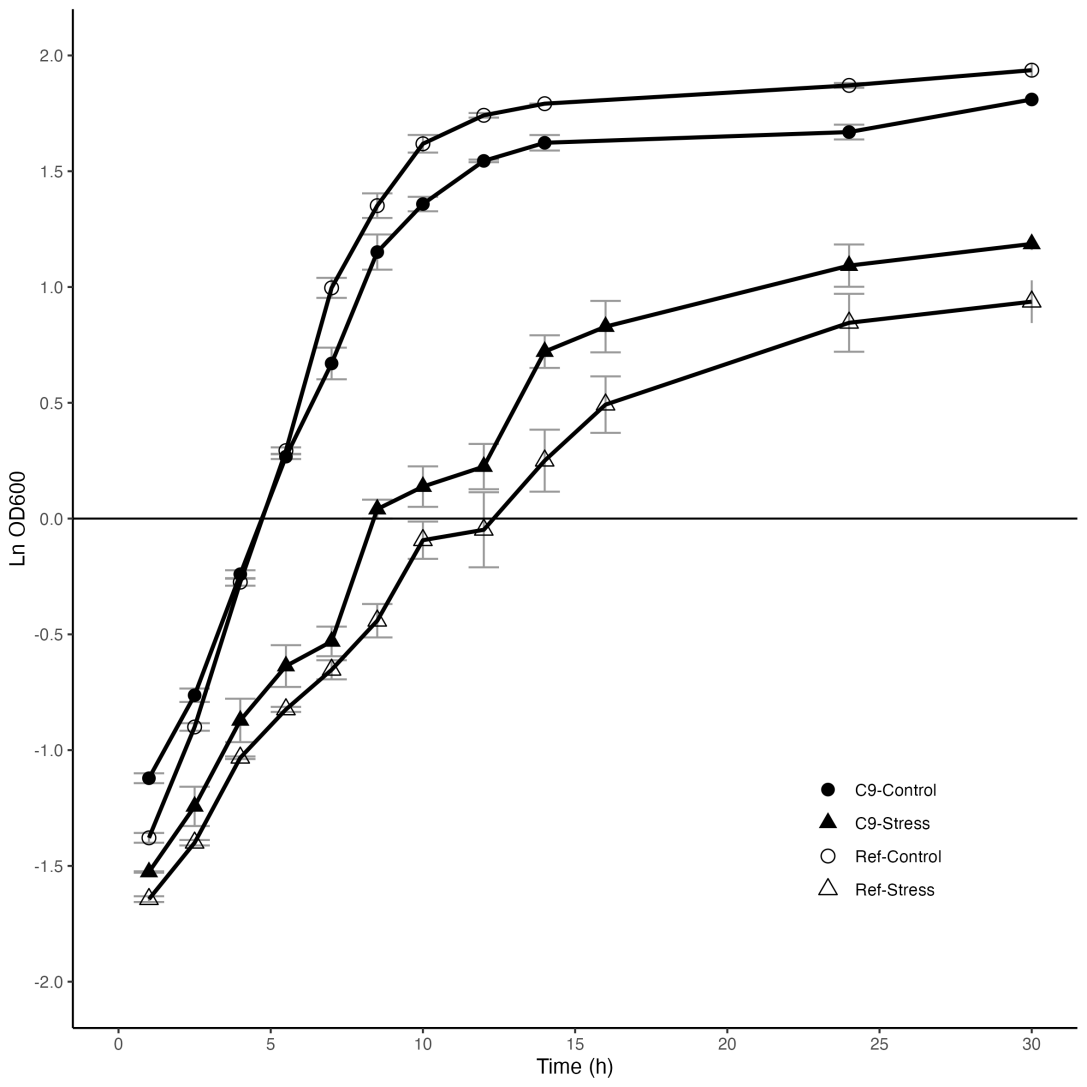
Growth behavior of the reference strain (Ref) and the evolved strain C9 in the absence and presence of 3 g/L 2-phenylethanol stress.

Growth profiles on a 2% glucose synthetic medium showed that the maximum specific growth rate of the reference strain (0.42 h^−1^) was significantly higher than that of C9 (0.32 h^−1^). In the presence of 3 g/L 2-PE stress, the evolved strain C9 was, in agreement with our plates data (Supplementary Figure S1), growing better than the reference strain, exhibiting a maximum specific growth rate of 0.16 h^−1^, as compared to 0.13 h^−1^ for the reference strain (Figure 3).

**Figure 4:**
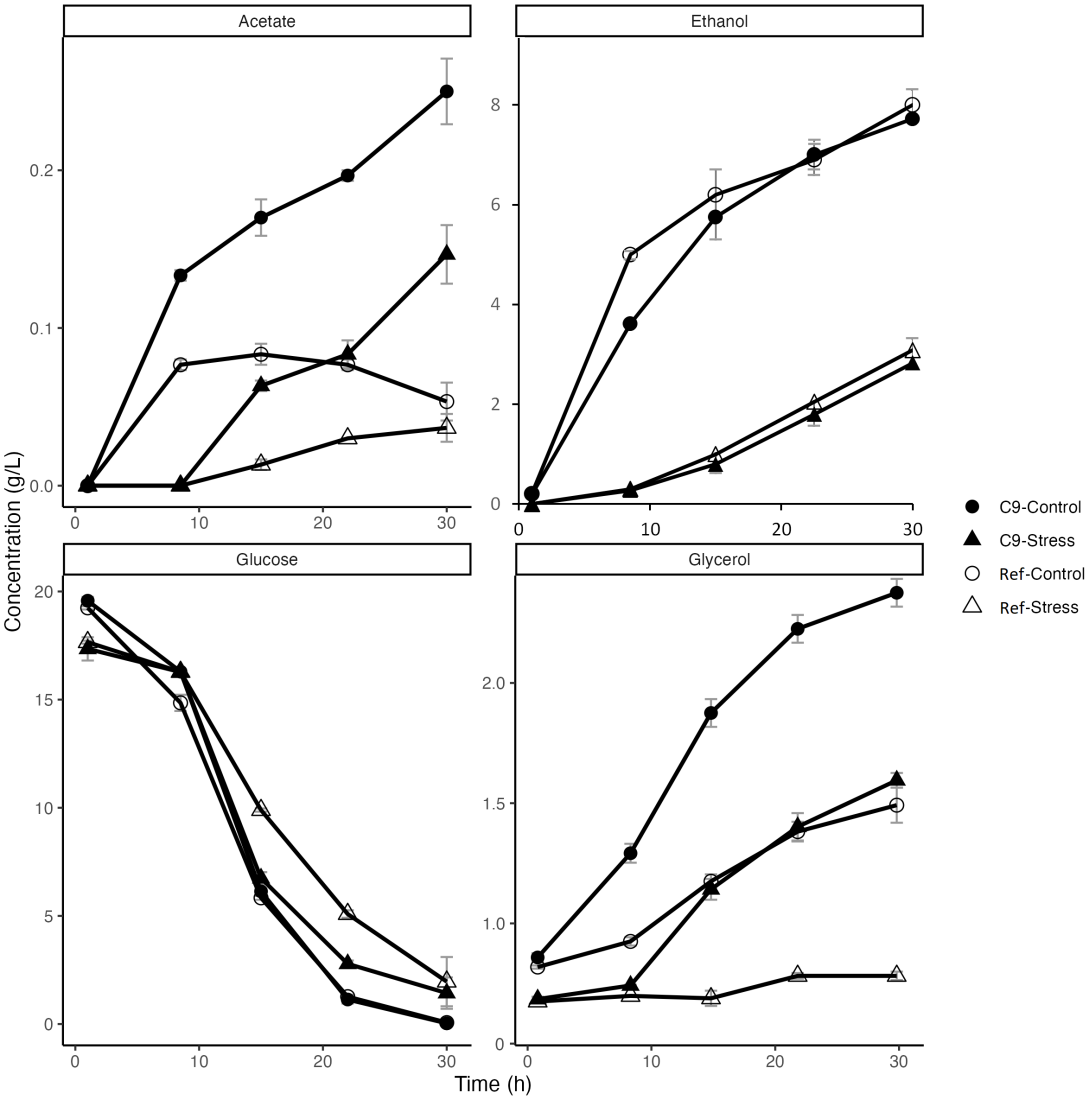
Metabolite profiles of the reference strain (Ref) and the evolved strain C9 in the absence and presence of 3 g/L 2-phenylethanol stress.

The metabolite profiles of C9 and the reference strain are presented in Figure 4. Both strains showed similar characteristics for glucose consumption and ethanol production in the absence and presence of 3 g/L of 2-PE stress. At the end of the cultivation (30th hour), glucose was depleted in the culture with both strains, while the produced ethanol concentration reached 8 g/L in the absence and 3 g/L in the presence of 3 g/L of 2-PE. This reduced production of ethanol suggested that the presence of 2-PE has modified the fermentation profile of yeast, leading to other by-products. Accordingly, the production of other fermentation by-products acetate and glycerol at the end of the glucose consumption significantly increased in C9 up to about 6-fold of the reference strain under control conditions. In the presence of 3 g/L 2-PE stress, the levels of these exometabolites were still significantly higher in C9, compared to the reference strain (Figure 4).

**Figure 5:**
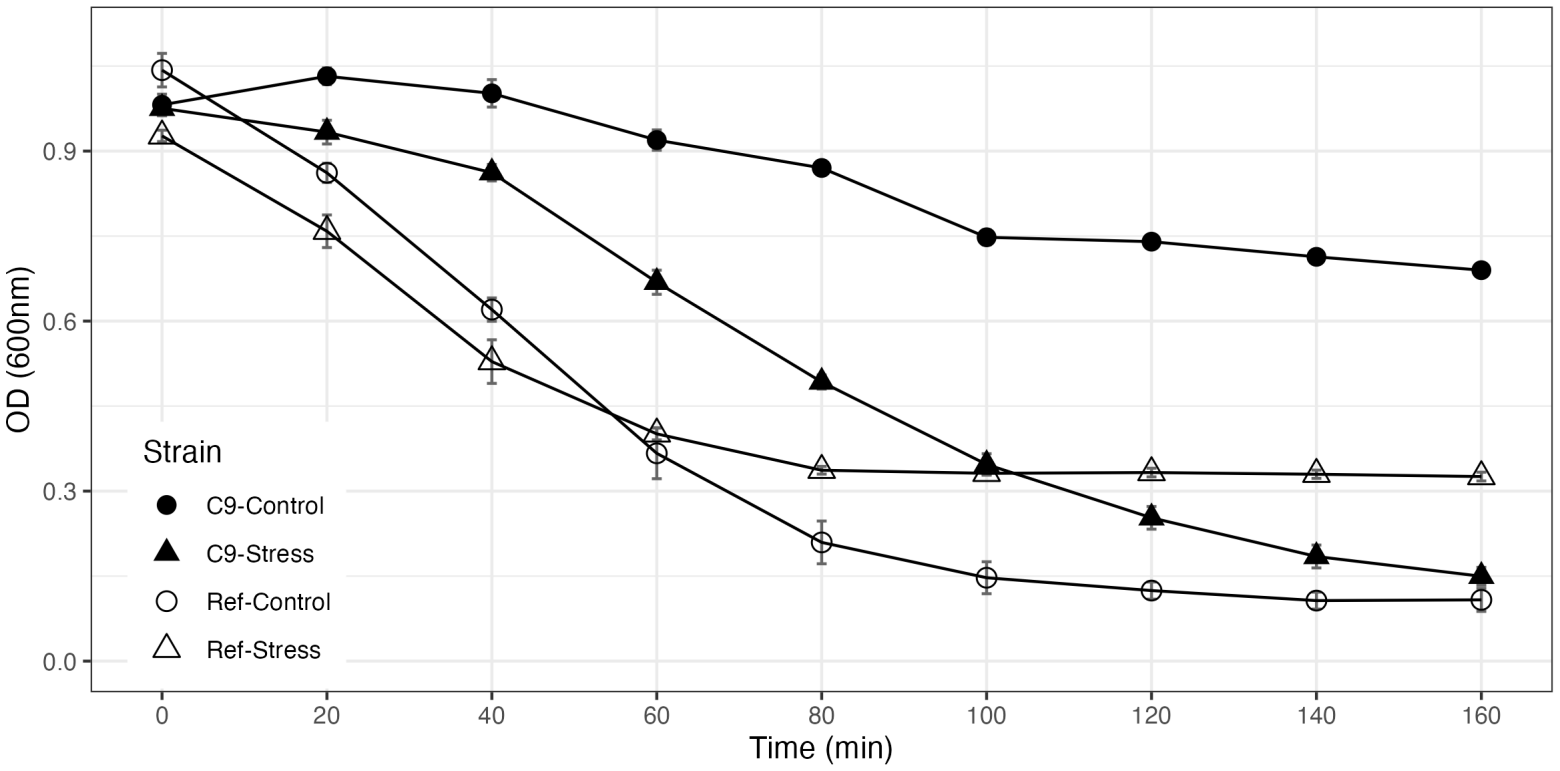
Lyticase sensitivity assay results of the reference strain (Ref) and the evolved strain under 3 g/L 2-phenylethanol stress and control conditions. Lyticase sensitivity was calculated as the percent decrease in lyticase resistance from 100% (the initial resistance value).

Of note, the levels of glycerol in C9 strain cultivated with and without 3 g/L 3-PE reached 1.5 and 2.4 g/L, respectively, which is up to about 3-fold higher than the levels of glycerol measured during growth of the reference strain, under the same conditions. In addition, C9 contained 3 times more trehalose than the reference strain in the absence of 2-PE and this difference increased to 5-fold in the presence of 3 g/L 2-PE stress (Supplementary Figure S3). On the other hand, the glycogen content of both strains was not significantly different from each other both in the absence and presence of 2-PE (Supplementary Figure S4). In previous studies dedicated to evolve yeasts to become resistant to either caffeine (Sürmeli et al., 2019), iron (Balaban et al., 2020), silver (Terzioğlu et al., 2020), or oxidative stress (Kocaefe-Özşen et al., 2022), we reported a relationship between resistance to these stresses and cell wall remodeling. Therefore, we also examined whether the resistance to 2-PE entailed some cell wall remodeling by a treatment of the strains with lyticase, a cell wall-degrading enzymatic cocktail (Kuranda et al., 2006). Our results revealed that the evolved strain had a significantly higher resistance against lyticase than the reference strain (Figure 5), indicating that some cell wall remodeling had occurred in response to 2-PE adaptation.

### 3.4. Comparative genome sequencing of the evolved strain to the reference strain

Whole-genome sequencing of the evolved and the reference strain was performed using next-generation shotgun sequencing with Illumina 2-channel sequencing by synthesis (SBS) technology. Upon sequencing, about 20 million reads with an average length of 270 bp were produced for the evolved C9 strain and the reference strain, which corresponded to 205x and 224x genome coverage, respectively. As we did not detect any indels in the genome sequence, we focused our analysis on SNPs and found 53 single nucleotide variations (SNVs) in the C9 genome, including 3 nonsense, 28 nonsynonymous, 7 synonymous, and 15 intergenic mutations. In line with the EMS mechanism of action, 50 of these mutations were transition mutations (C > T, G > A, or T > C) and the remaining three were transversion mutations (C > G or T > A). The list of the mutations detected only in coding sequence are reported in Table 1.

**Table 1:** The mutations detected in 2-phenylethanol-resistant mutant strain.

Gene	Genomic location	Amino acid change	Type	Description (Cherry et al., 2012)
*AAC1*	chrXIII:381787C > T	p.G182S	Missense variant	Mitochondrial inner membrane ADP/ATP translocator
*BNI1*	chrXIV:125713C > T	p.A1177T	Missense variant	Formin
*CRH1*	chrVII:877252C > T	p.D223N	Missense variant	Chitin transglycosylase
*DCN1*	chrXII:384270 G > A	p.E65K	Missense variant	Scaffold-type E3 ligase
*DON1*	chrIV:1009752 G > A	p.E312K	Missense variant	Meiosis-specific component of the spindle pole body
*DSS1*	chrXIII:837586C > T	p.R769K	Missense variant	3′-5′ exoribonuclease
*FLR1*	chrII:253415 G > A	p.P161S	Missense variant	Plasma membrane transporter of the major facilitator superfamily
*FMP30*	chrXVI:358453C > T	p.G301D	Missense variant	Protein with a role in maintaining mitochondrial morphology
*GAL80*	chrXIII:178921C > T	p.H376Y	Missense variant	Transcriptional regulator involved in the repression of GAL genes
*HFM1*	chrVII:29474C > T	p.R558Q	Missense variant	Meiosis specific DNA helicase
*HGH1*	chrVII:870124C > T	p.D340N	Missense variant	Chaperone protein for translation factor eEF2
*HOG1*	chrXII:357593 > G	p.F318L	Missense variant	Mitogen-activated protein kinase involved in osmoregulation
*MAK5*	chrII:522728 G > A	p.A215T	Missense variant	Essential nucleolar protein
*MCA1*	chrXV:717154 G > A	p.G105D	Missense variant	Ca^2+^ −dependent cysteine protease
*MMS2*	chrVII:346855C > T	p.S51N	Missense variant	Ubiquitin-conjugating enzyme variant
*MRPL32*	chrIII:118416 G > A	p.G108S	Missense variant	Mitochondrial ribosomal protein of the large subunit
*PDE2*	chrXV:1014250 G > A	p.P132S	Missense variant	High-affinity cyclic AMP phosphodiesterase
*PNT1*	chrXV:821568 G > A	p.G262D	Missense variant	Mitochondrial integral inner membrane protein
*POG1*	chrIX:123180C > T	p.Q291*	Stop gained	DNA-binding transcriptional activator
*POT1*	chrIX:32995C > T	p.G52D	Missense variant	3-ketoacyl-CoA thiolase with broad chain length specificity
*RPN11*	chrVI:149822 G > A	p.G99S	Missense variant	Metalloprotease subunit of 19S regulatory particle
*RPN4*	chrIV:414058 T > C	p.K504E	Missense variant	Transcription factor that stimulates expression of proteasome genes
*SFB3*	chrVIII:292692C > T	p.R805K	Missense variant	Component of the Sec23p-Sfb3p heterodimer of the COPII vesicle coat
*SPA2*	chrXII:87941 G > A	p.A658T	Missense variant	Component of the polarisome
*SSK2*	chrXIV:675235 T > A	p.M1316L	Missense variant	MAP kinase kinase kinase of HOG1 mitogen-activated signaling pathway
*TAF2*	chrIII:200518 T > A	p.K1316*	Stop gained	TFIID subunit (150 kDa)
*URN1*	chrXVI:832562C > T	p.A298T	Missense variant	Protein of unknown function containing WW and FF domains
*XRS2*	chrIV:1213976 G > A	p.R753C	Missense variant	FHA domain-containing component of the Mre11 complex

The mutations in intergenic regions and hypothetical genes were removed.

Two of them were in the *HOG1* and *SSK2* genes that belong to the mitogen-activated protein kinase involved in osmoregulation (Hohmann, 2002). The mutation in *HOG1* gave rise to a variant that converts phenylalanine to leucine at position 318 (Hog1p.F318L) exhibiting a hyperactive Hog1p kinase since this mutation is localized in the phosphorylation lip of this protein kinase (Bell et al., 2001). In favor of the increased activity resulting from this *HOG1* mutation, it was found that the evolved strain produced significantly higher amounts of glycerol (2.4 g/L) than the reference strain, in the absence of 2-PE (Figure 4). The mutation in the *SSK2* gene, which encodes the MAP kinase kinase kinase, upstream of Hog1p is located in the vicinity of the active site of this kinase and thus may lead to its loss of activity. As the mutation of *HOG1* gene already resulted in a hyperactive osmotic pathway, the Ssk2p.M1316L variant may be relevant for the alternative -*HOG1*-independent function of this kinase in mediating a calcium sensitivity response (Laviña et al., 2014) that we actually did not check in this study. Also relevant in regard to the physiological studies of the C9 strain was the finding of a mutation in *CRH1* that encodes a chitin transglycosidase responsible for chitin to β-1,6 glucan cross-linking (Rodriguez-Pena et al., 2000). This mutation, which gave rise to the Crh1p.D223N variant, is located in the linker region between sugar-binding areas of this enzyme (Blanco et al., 2015). While the effect of this mutation on the protein structure and enzyme activity remains to be clarified, it is possible that the finding of a higher resistance of C9 cells to lyticase, which has β-1,3-glucan hydrolase and β-1,3-glucanase activity, resulted from the expression of this Crh1p.D223N variant. Additionally, two missense mutations were found in *BNI1* (Bni1p.A1177T) and *SPA2* (Spa2p.A658T) that encode components of the polarisome, which is required for the proper initiation of bud growth and the proper shape of the bud. The mutation in these two genes may contribute to protect cell from lysis during budding by some local cell wall remodeling machinery since this event is a highly vulnerable process and prone to cause lysis (Mishra et al., 2022).

Other unexpected mutations were also identified in the genome analysis of C9 strain. A missense mutation in *GAL80* encoding the transcriptional regulator of *GAL* genes leading to the Gal80p.H376Y variant has never been mapped but this mutation is located in a C-terminal domain of Gal80p where other mutations have been shown to lose the interaction with Gal4p (Pilauri et al., 2005). The *PDE2* gene encoding the high-affinity cyclic AMP phosphodiesterase (Sass et al., 1986) presents a mutation in the N-terminal domain of the protein (Pde2p.P132S) whose effect is not known, either. Other intriguing mutations were found such as in *FLR1* (Flr1p.P161S) that encodes an efflux transporter of the major facilitator superfamily, and in *MAK5* (Mak5p.A215T) that encodes a putative RNA helicase involved in pre-RNA processing and in large ribosomal subunit biogenesis. SNPs in genes that are implicated in DNA replication and repair (*DON1*, *HFM1*, *XRS2*) were also identified. Finally, mutations in genes of the ubiquitin-proteasome system, namely *MMS2* (Mms2p.S51N) and *RPN4* (Rpn4p.K504E) which, together with a mutation in *MCA1* encoding a homolog to human caspase (Madeo et al., 2002), could indicate some deficiency in clearance of misfolded and aggregated proteins by proteasome in the 2-PE-adapted strain. Altogether, these mutations support the notion that the toxicity of 2-PE in yeast is complex, triggering genetic modifications that are not trivial at first glance but some of them are likely linked to protect cell from this toxic compound such as through activation of the *HOG1*-dependent pathway.

### 3.5. Comparative transcriptomic analysis of the evolved strain with the reference strain

**Figure 6:**
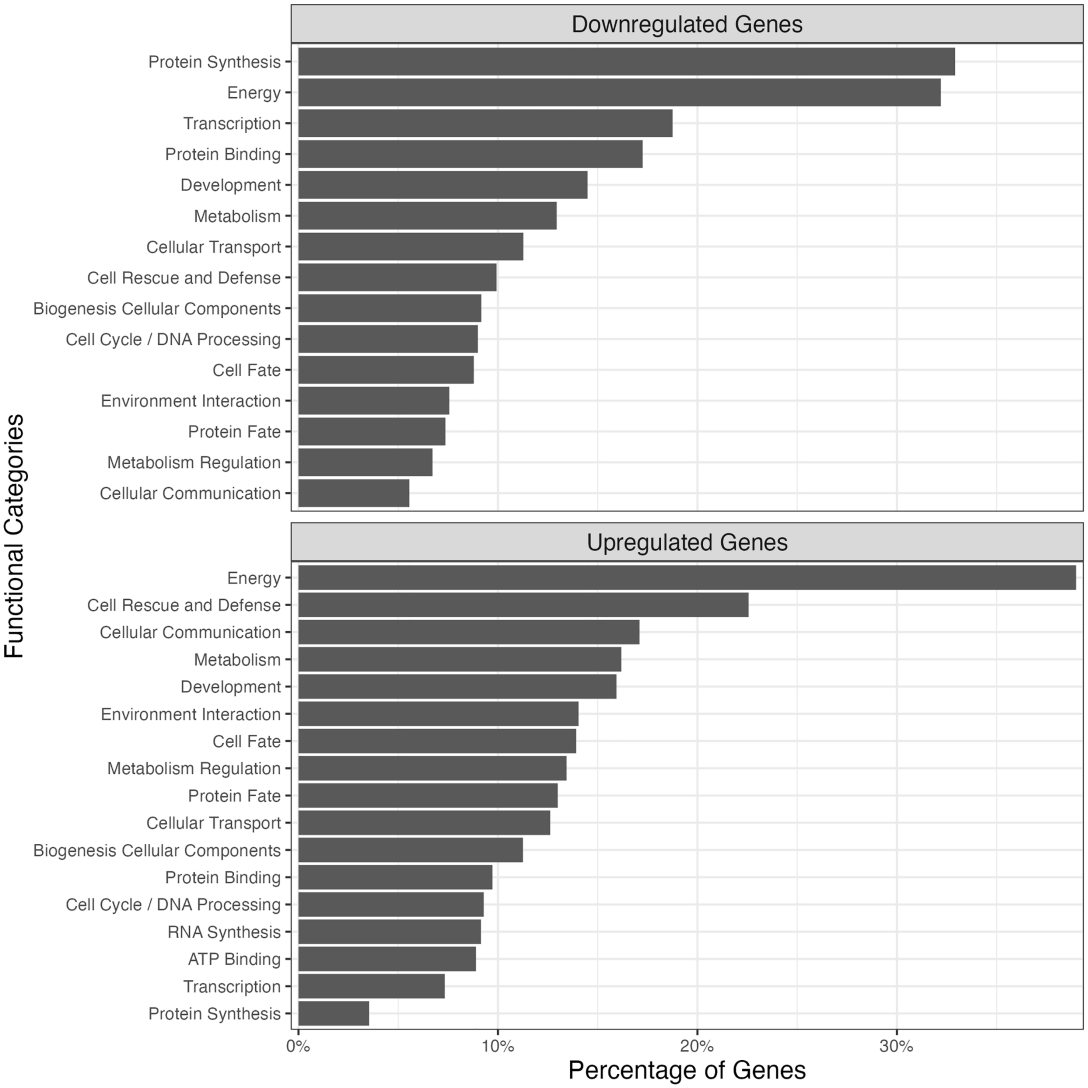
Functional categories and the corresponding percentages of the number of involved upregulated/downregulated genes of C9 among the total number of genes found in each functional category. The functional category annotation and gene clustering were performed using the FunCat database.

To complement the genome sequencing of the 2-PE-adapted strain, we performed a comparative transcriptomic analysis of this evolved strain with the reference strain to infer potential genetic targets of 2-PE-induced toxicity. This transcriptomic analysis showed that about 30% of all genes in the genome of C9 strain was differentially expressed with about 1,000 genes (17%) upregulated and 800 genes (13%) downregulated when a two-fold change and adjusted *p* values <0.05 were used as criteria for differential gene expression. Gene clustering using the FunCat database (Figure 6) showed that metabolic energy (39%) and cell rescue and defense (22%) were the most enriched functional categories among upregulated genes, whereas protein synthesis (33%) as well as energy (32%) were the major functional categories identified in the downregulated genes. Many genes associated with ATP production were also upregulated in the evolved strain, according to KEGG pathway analysis of the Oxidative Phosphorylation Pathway (KEGG ID sce00190; Supplementary Figure S5). Genes encoding several subunits of membrane proteins were upregulated in C9, including the SDHC and SDHA subunits of succinate dehydrogenase, *CYT1*, *COR1*, and *QCR7* subunits of cytochrome reductase, and *COX1, COX2, COX3, COX4,* and *COX6A* subunits of cytochrome c oxidase (Supplementary Figure S5). Furthermore, the alpha (*ATP1*), beta (*ATP2*), epsilon (*ATP15*), a (*ATP6*), and j (*ATP18*) subunits of ATP synthase were significantly upregulated (Supplementary Table S2), suggesting that the 2-PE resistance may involve ATP-related activities in the oxidative phosphorylation pathway.

Gene ontology analysis of these transcriptomic data was also carried out using FUNspec.^4^ This analysis showed that the most enriched biological processes encompassing upregulated genes were response to stress, including the cellular response to oxidative stress and fungal cell wall organization (Table 2), whereas a large number of downregulated genes belonged to biological processes related to ribosomal biogenesis, translation and RNA processing (Table 3).

**Table 2:** FUNSpec Gene Ontology (GO) analysis results of the upregulated genes in C9 (*k*: number of genes in input cluster in given category, *f*: total number of genes in given category).

GO biological process			
Category	In category from cluster	*k*	*f*
Response to stress [GO:0006950]	*SSA1 FRT2 PAU8 PAU7 PAU9 HSP26 TPS1 SSE2 PAU24 PAU3 GPD1 MRK1 UFD2 FMP45 NTH1 TPS2 HSP42 HSP78 PAU10 PAU2 HOR2 SSA4 HSP12 PAU5 MTL1 CTT1 FMP43 PAU12 PAU13 GRE3 XBP1 PAU14 PAU15 PAU1 DAN4 MSN4 MNN4 PAU16 PAU17 HSP104 UBI4 PAU18 YLR040C ICT1 DAK1 TSL1 RIM11 DDR48 HOR7 TPS3 PAU19 OCA1 MDJ2 PAU6 DDR2 RSB1 HSP33 PAU21 GRE1 HSP82 HSP32 PAU22 HAL1 ATH1*	64	152
Carbohydrate metabolic process [GO:0005975]	*GAL10 YBR056W MAL32 GLK1 GPD1 EMI2 GLC3 PCM1 GLC7 HXK1 AMS1 NQM1 CRH1 XKS1 SOL4 SCW4 BGL2 IMA1 MAL12 YHR210C PKP1 SGA1 YIR007W IMA5 MDH1 CTS1 YLR446W PGM2 PGM3 CAT8 SOL1 MDH2 GAL4 ATH1 GPH1 GDB1*	36	94
Cellular response to oxidative stress [GO:0034599]	*PRX1 UGA2 GRX7 ZTA1 YBP1 GRX1 GRX6 YDL124W YPR1 TSA2 GRX2 HSP12 MTL1 TRX2 GRE3 TRR2 YJR096W GPX1 MSN4 SRX1 MCR1 AHP1 GAD1 NCE103 OCA1 GCY1 OXR1*	27	67
Maltose metabolic process [GO:0000023]	*MAL31 MAL32 MPH2 IMA1 MAL13 MAL11 MAL12 IMA5 MPH3*	9	11
Carbohydrate transport [GO:0008643]	*MAL31 SNF3 MPH2 HXT7 HXT6 HXT3 STL1 HVG1 MAL11 HXT1 HXT5 HXT8 MPH3 GAL2 HXT11*	15	31
Trehalose biosynthetic process [GO:0005992]	*TPS1 TPS2 UGP1 TSL1 PGM2 TPS3*	6	7
Glycogen biosynthetic process [GO:0005978]	*GLC3 GSY1 UGP1 GLG1 GSY2 PGM2 GAC1 GDB1*	8	12
Glycolysis [GO:0006096]	*GLK1 GPM2 EMI2 HXK1 ENO1 TDH1 FBA1 YLR446W ERR3 PYK2 ERR1 ERR2*	12	28
Oxidation–reduction process [GO:0055114]	*COX1 COX2 COX3 BDH1 BDH2 PRX1 UGA2 ETR1 ZTA1 ARA1 HBN1 GPD1 NDE2 YDL124W GDH2 PAM1 YPR1 TSA2 SER3 MET13 ARI1 AIM14 CTT1 COQ6 GND2 AIM17 SOD2 HTD2 GRE3 TRR2 COX5B GUT2 TDH1 YJR096W GPX1 MDH1 SRX1 YKL107W SDH1 MCR1 FMP46 AHP1 IDP2 HMX1 CYB2 NDI1 YML131W FMS1 HFD1 ALD3 ALD2 GOR1 ADH1 MDH2 GRE2 GCY1 ALD4 FDH1 IRC15 ALD6 YPL088W OYE3 YPR127W*	63	272
Phosphorylation [GO:0016310]	*PSK1 CDC15 TEL1 AKL1 VPS15 GLK1 KIN82 RTK1 MRK1 PRR2 KIN1 ARG82 VHS1 IPK1 PKH3 PKH1 EMI2 CMK1 FAB1 HXK1 XKS1 TDA10 SLT2 PKP1 PFK26 IKS1 YAK1 TPK1 UTR1 PTK2 YNK1 KDX1 KKQ8 NNK1 KNS1 YLR345W YLR446W DAK1 YPK2 RIM11 TDA1 ARK1 FPK1 PKH2 PYK2 MEK1 FRK1 TPK2*	48	206
Fungal-type cell wall organization [GO:0031505]	*ECM8 FMP45 PST1 SED1 ZRG8 MPT5 MTL1 ROM1 SKN1 CRH1 BGL2 ECM12 SLT2 SIM1 MHP1 TAX4 CIS3 ECM27 CWP1 CWP2 MYO3 PIR3 ECM4 CCW12 YPS1 YPS3 ECM30 AVO2 KRE1 SRL1 RLM1*	31	128

**Table 3:** FUNSpec Gene Ontology (GO) analysis results of the downregulated genes in C9.

GO Biological Process				
Category	*p*-value	In category from cluster	*k*	*f*
Ribosomal large subunit biogenesis (GO:0042273]	1.00E-14	*MAK16 MAK5 REI1 YDL063C RLI1 ARX1 PUF6 NOP16 NSA2 NSA1 SDA1 CIC1 ALB1 MRT4 MAK11 RIX7 RLP24 SDO1 AFG2 ERB1 RLP7 NOP15 NOP8 YTM1 RRS1 NOG1 NIP7 TIF6*	28	37
Maturation of SSU-rRNA from tricistronic rRNA transcript (SSU-rRNA, 5.8S rRNA, LSU-rRNA) [GO:0000462]	1.00E-14	*RPS11B RPS6B RPS9B RPS14A PWP2 RPS16B SAS10 FAP7 FAL1 RPS11A RPS13 UTP4 UTP5 UTP6 SNU13 RPS8B SLX9 UTP22 UTP8 EFG1 RPS27B UTP9 FAF1 UTP25 UTP10 DHR2 RPS27A SOF1 FYV7 DIP2 HCR1 UTP13 TSR2 RPS1A RPS1B ECM16 RPS16A RIO2 NOB1 RRP36 RRP12 NAN1*	42	60
Ribosome biogenesis [GO:0042254]	1.00E-14	*MAK5 RPS6B RPS9B ENP1 REI1 SPB1 RPS14A PWP2 NOP1 TSR1 SAS10 FAP7 FAL1 MAK21 RLI1 SSF2 UTP4 FCF1 ESF1 UTP5 UTP6 SNU13 NOP16 NUG1 NSA2 LCP5 SPB4 MRH4 NSA1 SLX9 UTP22 UTP8 ENP2 SDA1 RNH70 BCD1 CIC1 SSF1 NOP10 IPI1 RPF1 IMP3 DBP8 UTP9 RIX1 FAF1 UTP25 NOP9 HCA4 UTP18 UTP10 ALB1 MPP10 URB2 MRT4 MAK11 DHR2 UTP11 EBP2 DBP7 UTP30 RPL40B DRS1 SOF1 RIX7 RPL8B NOC3 RLP24 SDO1 RPSOB FCF2 DIP2 EMG1 UTP13 DBP9 UTP21 UTP14 ERB1 ECM16 RRB1 RRP5 HAS1 RLP7 NOP2 IMP4 NOP15 DBP2 NAF1 IPI3 RIO2 KRI1 DBP6 NOG2 ESF2 RCL1 NOP12 BRX1 NOP8 UTP23 NOB1 RPS7A PNO1 NOC2 PUS7 YTM1 RRP36 RRS1 RRP12 NOG1 NAN1 NOP53 RTC6 RSA1 NIP7 RRP9 RRP15 NOC4*	117	170
rRNA processing [GO:0006364]	1.00E-14	*MAK5 RPS6B RPS9B ENP1 POP4 SPB1 RPS14A PWP2 NOP1 TSR1 RRP42 SAS10 FAP7 SSB1 FAL1 RRP8 RRP1 RLI1 RRP45 BFR2 UTP4 FCF1 ESF1 UTP5 UTP6 SNU13 NOP16 NUG1 NSA2 LCP5 SPB4 RPL30 NSA1 RAI1 POP6 SLX9 UTP22 RRP46 UTP8 ENP2 MTR3 NSR1 EFG1 RNH70 ZUO1 RPP1 RRP4 NOP10 IPI1 RPF1 IMP3 DBP8 UTP9 RIX1 UTP25 NOP9 HCA4 MTR4 UTP18 UTP10 MPP10 URB2 MRT4 DHR2 UTP11 EBP2 DBP7 UTP30 DRS1 SOF1 GRC3 NOC3 SDO1 RPSOB FCF2 FYV7 DIP2 EMG1 PWP1 UTP13 DBP9 UTP21 TSR2 UTP14 ERB1 ECM16 RRB1 RRP5 RNT1 HAS1 NOP2 IMP4 NOP15 DBP2 NAF1 IPI3 SSB2 CSL4 KRI1 DBP6 ESF2 DIS3 TSR4 NOP12 REX4 RRP40 UTP23 RPS7A PUS7 YTM1 RRP36 NOG1 NAN1 NOP53 NIP7 TIF6 MRD1 RRP9 RRP15 NOC4*	120	195
Translation [GO:0006412]	1.25E-09	*MAK16 FUN12 RPL19B RPL23A RPS11B TRM7 RPG1 RPL19A MRPS9 RPS6B RPS9B RPL21A YCL001W-B RPS14A RPP1A RPL13A RPS16B RPL41B RPL41A SSB1 RPL4B RPS11A RPS13 RLI1 GIR2 MRPL7 RPP2B TIF35 RPS18A RPL27B RPL12A RPS8B RPS26B RPL30 RPL28 RPL1B YGR054W RBG2 YHR020W RPS27B RPF1 RPL42B MRPL6 RPS4B RPL34B RPL16A MRPL8 MRPL49 RPL17B RPS22A SUI2 ANB1 RPL14A RPS27A GCN3 RPL40B RPL8B RLP24 RPSOB FRS1 HCR1 RPS28B RPS25B RPL26A RPS1A RPS17A RPS1B RPS16A TIF34 MRPL33 RLP7 MRP7 RPL16B IMP4 RPL42A MRPL19 SSB2 SUI1 RPS19B BRX1 WRS1 RPS19A RPL25 CDC33 TMA46 RPS7A GCD1 CAF20 RPS10A RPS12 RPL21B RPL33A RTC6 RPL1A SUI3 TIF6 TIF5*	97	318
RNA processing [GO:0006396]	9.80E-05	*LHP1 RRP42 TRM3 RRP45 UTP6 RPL1B RRP46 MTR3 CIC1 TRM2 UTP30 FYV7 PRP39 RNA14 RRP5 RNT1 PUS4 RCL1 MRM1 RPL1A*	20	37
tRNA processing [GO:0008033]	1.23E-04	*SEN34 TRM7 POP4 NOP1 LHP1 TRM3 TRM8 TRM1 TRM82 SUA5 MT01 POP6 PUS6 RNH70 RPP1 TRM5 URM1 TRM2 TRZ1 TAD3 DUS3 TRM12 DUS1 GCD10 NCS2 PUS4 SMM1 TRM10 TRM13 PUS7 TRM44*	31	80
Maturation of LSU-rRNA from tricistronic rRNA transcript (SSU-rRNA, 5.8S rRNA, LSU-rRNA) [GO:0000463]	6.18E-04	*MAK16 MAK5 SPB1 NSA2 RAI1 RPF1 MAK11 RPF2 RLP7 NOP12 NOP53 TIF6 RRP15*	13	18
Nuclear polyadenylation-dependent tRNA catabolic process [GO:0071038]	1.22E-03	*RRP42 RRP45 RRP46 MTR3 RRP4 AIR1 MTR4 CSL4 TRF5 DIS3 PAP2 RRP40*	12	16
tRNA transcription from RNA polymerase III promoter [GO:0042797]	7.60E-04	*RPB5 RPC53 RPC17 RPC25 RPC37 RPC19 RPC31 RPC34 RPC40 RPO26 RPC82*	11	15
Ribosomal subunit export from nucleus [GO:0000054]	1.48E-02	*SSB1 RLI1 LSG1 ZUO1 RIX1 SDO1 SSB2 NOC2 NOG1 TIF6*	10	13
Nuclear polyadenylation-dependent rRNA catabolic process [GO:0071035]	2.16E-02	*RRP45 RRP46 MTR3 RRP4 AIR1 MTR4 CSL4 TRF5 DIS3 PAP2 RRP40*	11	16
Maturation of 5.8S rRNA from tricistronic rRNA transcript (SSU-rRNA, 5.8S rRNA, LSU-rRNA) [GO:0000466]	4.61E-02	*MAK16 MAK5 SPB1 NSA2 RAI1 RPF1 MAK11 RPF2 TIF6 RRP15*	10	14
Pyridoxine biosynthetic process [GO:0008615]	1.78E-01	*BUD16 SNZ3 SNO3 SNO1 SNZ1 SNZ2 SNO2 RKI1*	8	10
Pyrimidine nucleotide biosynthetic process [GO:0006221]	5.83E-01	*URA7 URA3 DCD1 URA6 URA1 PPR1 URA4 URA5*	8	11
Transmembrane transport [GO:0055085]	1.16E+00	*SEO1 FUI1 FUR4 BAP2 TAT1 YMC2 DTR1 CTP1 VBA2 SUL1 GEX1 GIT1 GGC1 UGA4 ENA2 BAP3 VBA4 ATO3 FCY2 KAP123 FTR1 HNM1 ZRT1 MUP1 VHT1 MEP1 YGR125W HIP1 TNA1 YHK8 POR2 QDR1 PHO90 HXT9 YJR124C MCH2 GAP1 GEX2 MMP1 PAM18 YML018C NDC1 ATR1 PHO84 VBA1 ZRC1 FET4 AQR1 YNL095C MEP2 ALP1 TIM23 BIO5 HXT17 TAT2 ENB1 YOL162W YOL163W MCH5 SSU1 HUT1 MEP3 OPT2*	63	303
Cellular amino acid biosynthetic process [GO:0008652]	3.05E+00	*MET8 HIS7 ARO4 CTR86 AR03 LYS4 PRO1 HIS1 ILV1 MET6 IRC7 ARO2 SER2 HIS6 LYS1 ILV3 MHT1 YML082W YML096W ADI1 ARG8 LEU9 HIS3 SAM4 AR07 MRI1 ASN1*	27	98

*k*: number of genes in input cluster in given category, *f*: total number of genes in given category.

**Figure 7:**
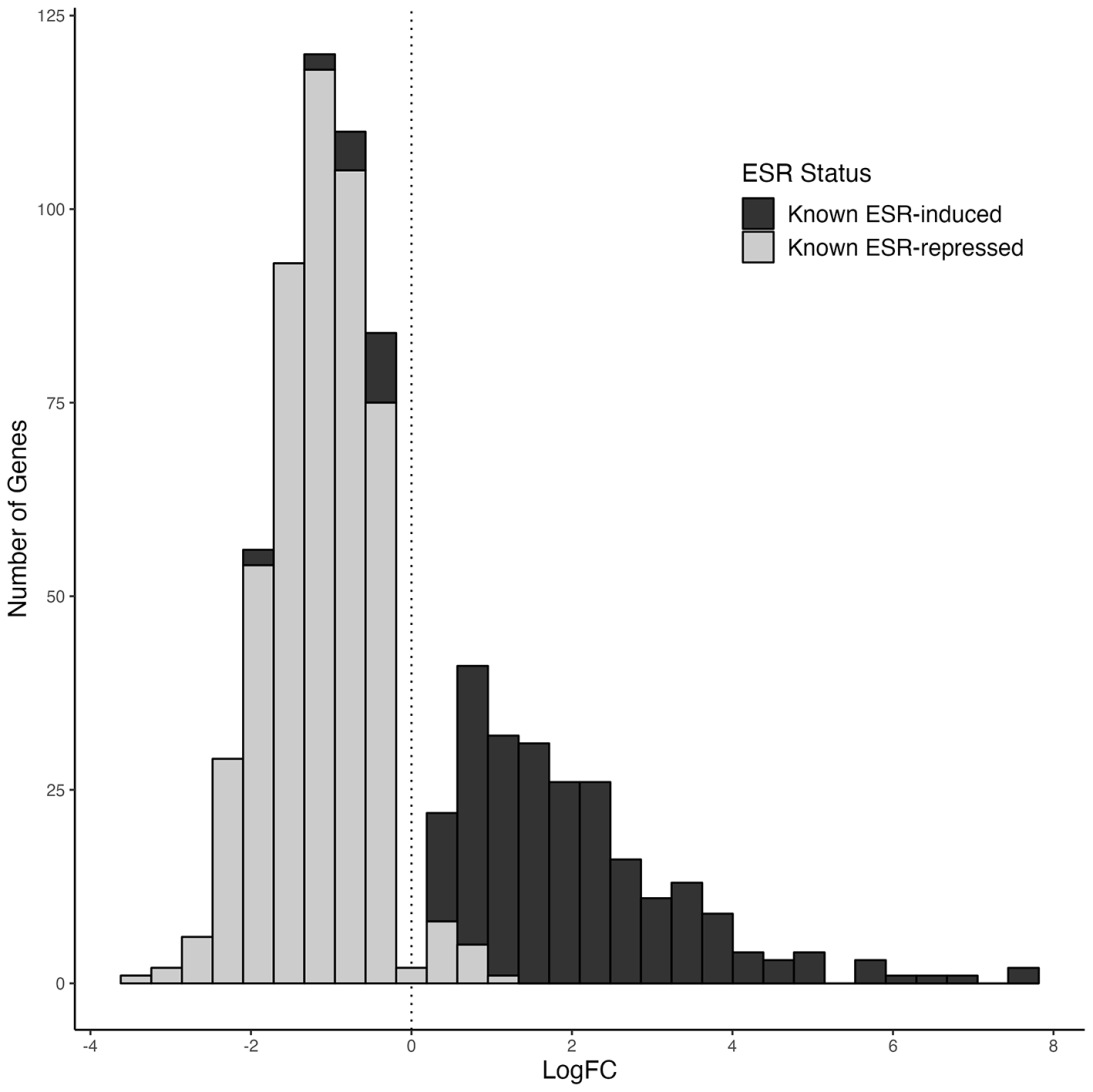
Expression distribution of known environmental stress-response (ESR) gene groups in the 2-phenylethanol-resistant strain C9. The gray columns show the previously known genes that are expected to be repressed under ESR, while the black columns show the previously known genes that are expected to be induced under ESR, according to Brauer et al. (2008).

Altogether, these data indicated that the 2-PE adapted C9 strain is set in a so-called stressing state. These data are supported by the finding that 93.9% of the upregulated genes and 96.4% of the downregulated genes in this C9 strain belong to the environmental stress response (ESR) reported in Brauer et al. (2008) (Figure 7).

These transcriptomic data were also analyzed using the YEASTRACT database,^5^ in particular to determine which transcription factors are implicated in the expression of enriched categories of upregulated and downregulated genes. As expected, Msn2p/Msn4p and Yap1p, which are known to induce expression of many stress-regulated genes were recovered in about 67 and 51% of all upregulated genes, respectively, with hallmarks genes that belong to glycogen and trehalose biosynthesis and stress response (Table 4). The transcription factors Sok2p and Ste12p were also found. They both participate in regulating genes involved in pseudo-hyphae/invasive growth through a MAP kinase pathway (Pan and Heitman, 2000; Malcher et al., 2011).

**Table 4:** Transcription factors and their percent contribution to the upregulated genes that have regulatory interaction of 2-PE-evolved strain C9.

Transcription factor	Percent contribution
Msn2p and Msn4p	67.0 and 53.3%, respectively
Aft1p	59.4%
Rpn4p	57.1%
Ste12p	56.1%
Sok2p	54.7%
Yap1p	50.9%

Percent contribution shows the percentage of significantly changed genes that has regulatory interaction with a given transcription factor according to the YEASTRACT database.

More trivially, most of the stress-responsive genes harbor in their promoter a Sok2p and/or a Ste12p binding motif, as can be easily obtained through YEASTRACT analysis (data not shown). The transcription factors Rpn4p, which is reported as an activator of proteasome (Xie and Varshavsky, 2001) and Aft1p, which activates transcription in response to iron availability (Yamaguchi-Iwai et al., 1995) were also identified. Overall, the presence of these transcription factors are in concordance with the pathway enrichment analysis, that notably highlighted MAPK signaling, proteasome, autophagy and ribosome biogenesis (Table 5).

**Table 5:** The significantly (FDR adjusted *p* values <0.05) altered pathways according to the pathway enrichment analysis results of the evolved strain C9.

Pathway	Gene-set level change	Adjusted *p*-value	Genes
sce00500 Starch and sucrose metabolism	6.070	6.10e-06	41
sce00010 Glycolysis/Gluconeogenesis	3.654	1.05e-02	55
sce00052 Galactose metabolism	3.611	1.40e-02	24
sce04011 MAPK signaling pathway–yeast	3.272	1.40e-02	114
sce00561 Glycerolipid metabolism	3.202	2.11e-02	29
sce01200 Carbon metabolism	2.918	2.99e-02	114
sce04138 Autophagy–yeast	2.703	4.94e-02	85
sce03050 Proteasome	−3.114	2.94e-02	35
sce03013 RNA transport	−3.245	1.64e-02	85
sce03020 RNA polymerase	−5.050	1.15e-04	30
sce03008 Ribosome biogenesis in eukaryotes	−6.654	1.37e-08	73
sce03010 Ribosome	−9.309	4.06e-16	168

Pathway enrichment analyses were performed using the Limma package in R/Bioconductor, based on the KEGG pathway database.

**Figure 8:**
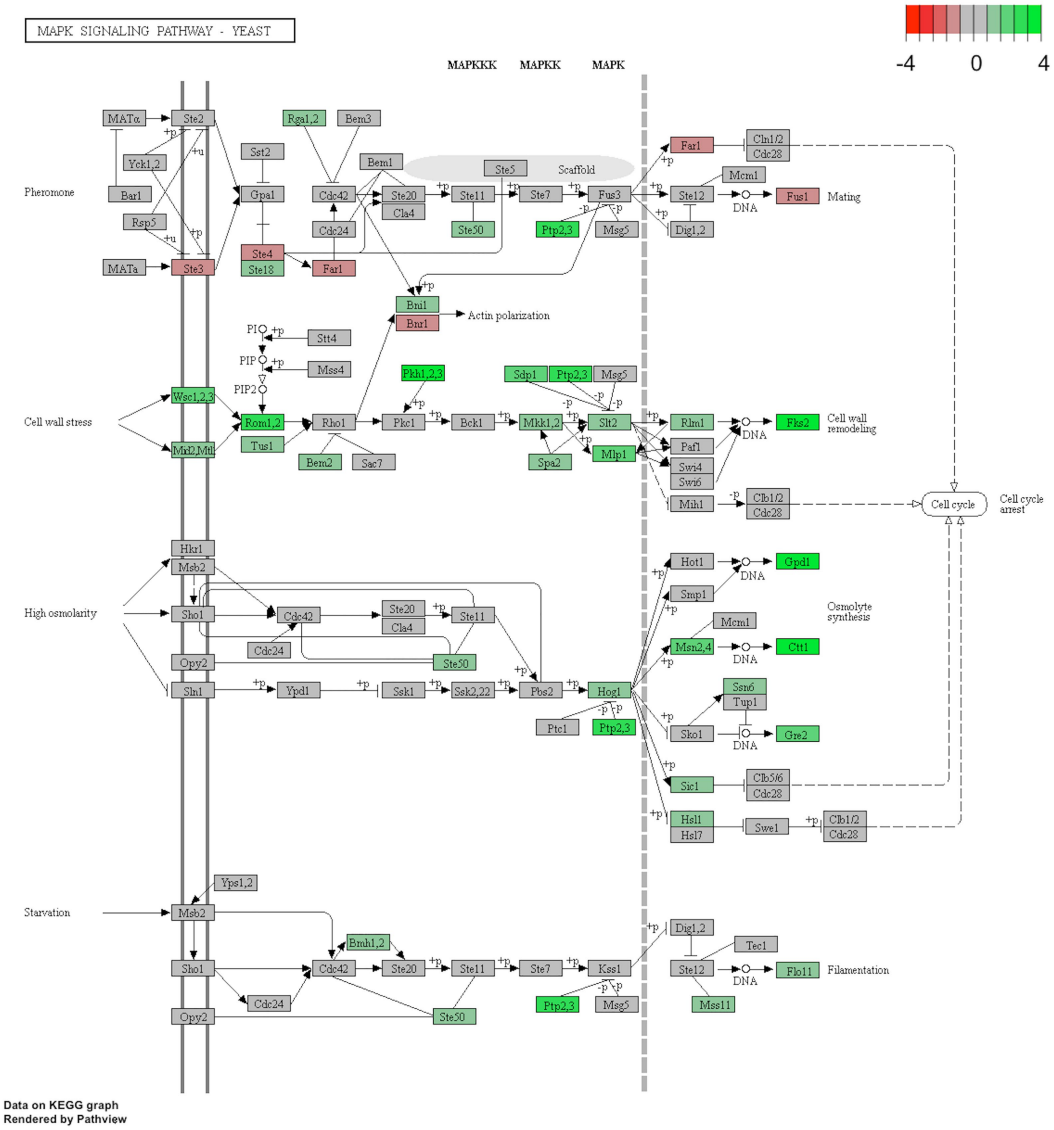
KEGG pathway of the MAPK Signalling Pathway (sce04011) that belongs to the 2-phenylethanol-resistant strain C9. Red boxes show the genes that were downregulated, while the green boxes show the upregulated genes.

In addition, KEGG pathway analysis of the MAPK signaling pathway showed that the differentially expressed genes in C9 were mainly related to cell wall remodeling, osmolyte synthesis, filamentation and mating (Figure 8).

In complement to this hierarchical analysis, we looked more closely to some genes that were either highly upregulated or downregulated in 2-PE-adapted strain C9 by a factor of at least 5-fold (Supplementary Tables S3, S4). Among these strongly upregulated genes, it is worth to notice genes involved in maltose metabolism including the maltose transporter (*MAL11*, *MAL31*) and maltase-encoding genes (*MAL12*, *MAL32*). This strong upregulation of these *MAL* genes could be caused either by their dependence on the transcriptional regulation by Msn2/4p and/or by their position at the telomeres of yeast chromosome VII (*MAL11*, *MAL12*, *MAL13*) and by chromosome II (*MAL31*, *MAL32* and *MAL33*). Several genes of the galactose metabolism, i.e., *GAL2*, *GAL10*, *GAL4,* and *PGM2*, were also found to be upregulated in C9 (Supplementary Table S3). The transcriptional increase of these genes could be linked to the *GAL80* missense mutation found in the C9 strain. Potent transcriptional increase was also noted for all the genes that belong to storage carbohydrates and many specific genes whose expression can be activated under various stress conditions such as *CTT1*, *DDR2*, *HSP12*, *GRE2*, etc. (see Supplementary Table S3; González et al., 2000). This potent upregulation can be explained by the presence in their promoter of one to several Msn2/Msn4p binding motifs termed STRE (Martinez-Pastor et al., 1996; Schmitt and McEntee, 1996). Another relevant finding was to detect a very potent transcriptional activation of *ALD3* (234-fold) and *ALD4* (28-fold) encoding promiscuous NAD^+^-dependent aldehyde dehydrogenases (Navarro-Aviño et al., 1999), as well as *BDH2* (21 fold), which codes for a putative medium-chain alcohol dehydrogenase with similarity to a butanediol dehydrogenase that oxidizes (R,R)-2,3-butanediol to (3R)-acetoin (González et al., 2000). The strong upregulation of these genes can also be explained by their transcriptional dependency on Msn2/Msn4p. Among the most highly downregulated genes, which included many of the genes encoding ribosomal proteins (Supplementary Table S4), there were also several genes related to the transport of inorganic phosphate (Pi), sulfate, ammonium, zinc, and iron, supporting the notion that 2-PE can interfere with membrane fluidization (Ingram and Buttke, 1984) and ion transport (Seward et al., 1996).

Finally, since C9 strain showed stronger resistance than the reference strain to degradation of its cell wall by lyticase, we inspected the differentially expressed genes that belong to cell wall organization. We found that expression levels of many cell wall mannoprotein-encoding genes (*CWP1*, *CWP2*, *FIT1*, *FIT2*, *CCW12*), genes involved in cell wall biogenesis (*ECM12*, *ECM27*, *ECM30*, *ECM4*, *ECM8*) as well as *CRH1* and *CRH2* encoding chitin transglycosylase that catalyzes the glycosyl linkage between chitin and β-glucans (Blanco et al., 2015) were increased by 3 to 5-fold in the C9 strain. The upregulation of the two latter genes may also complement the effect of the mutation in *CRH1* in increasing the resistance of the C9 strain to lyticase treatment. The upregulation of 20 out of the 24 genes of the *PAU* family can also be related to cell wall remodeling induced by 2-PE since *PAU* genes encode cell wall-related proteins, which are negatively regulated by oxygen and induced under hypoxic conditions (Rachidi et al., 2000; Alkim et al., 2013).

## 4. Discussion

In this work, the tolerance of the *S. cerevisiae* laboratory strain CEN.PK 113-7D to 2-PE was increased from 1.5 to 3.4 g/L by an adaptive laboratory evolution (ALE) strategy. This tolerance of the evolved strain was very similar to that obtained by a similar ALE strategy, except that in the latter, the adapted strain was forced to reach after 24 h the same optical density upon each additional increase of the 2-PE concentration (Zhu et al., 2021). While Zhu et al. (2021) did not report the genome sequencing of their evolved strain, the genome sequencing of our evolved strain revealed mutations in many genes that belong to various cellular functions and pathways, which at first glance, were not related to 2-PE detoxification or metabolization. On the other hand, the transcriptome of this evolved strain was profoundly altered with more than 30% of all genes in the genome being differentially expressed as compared to the original strain. In addition, the transcriptomic profile of our 2-PE-adapted strain very much resembled those of yeast challenged with various stresses and leading to the so-called ESR-induced and repressed genes (Gasch, 2000). These data strongly indicated that our C9 strain is poised in a permanent stressful state. Jin et al. (2018) reported on the transcriptomic response of *S. cerevisiae* upon exposure to 4 g/L 2-PE stress. At variance to our transcriptomic data, their data indicated that most of the highly upregulated genes reported in our study, notably in stress response, were found downregulated in their study (Jin et al., 2018). They also reported high enrichment in genes that belong to membrane and mitochondrial function, which was not the case in our work. Thus, there is a clear difference in the transcriptomic response of a strain challenged with 2-PE and that of a strain that has been adapted to this toxic compound. This difference may help to shed light on the mechanisms of resistance to 2-PE set up by the yeast, from those that were triggered in response to the presence of this toxic compound. Our study clearly showed that the adaptation to 2-PE led to a so-called stressful state of the cell.

The so called “stressful state” of our 2-PE-adapted strain C9 is likely the major consequence of two hierarchical events. At the genomic level, the mutations in the *HOG1* gene resulted in the expression of a hyperactive *HOG1*-dependent pathway, due to the fact that the mutation in this gene is located in the phosphorylation lip of Hog1p, which was previously reported to significantly increase the Hog1p kinase activity (Bell et al., 2001). This hyperactive Hog1p.F138L in turn activates a hyperosmotic stress resistance mechanism that was witnessed in our C9 strain by a significantly increased production of glycerol, as compared to the reference strain. Under osmotic stress conditions, yeast cells start to synthesize internal osmolytes, primarily glycerol, to create an osmotic gradient (Saito and Posas, 2012). The production of glycerol is mainly regulated by two genes in yeast: *HOG1* and *PBS2*. Null mutants of *HOG,* which encode a mitogen-activated protein kinase involved in osmoregulation, had decreased glycerol accumulation under osmotic stress (Brewster et al., 1993). It is also known that ethanol causes water stress in yeast by lowering water activity, and cells produce compatible solutes like glycerol and trehalose for their protection against dehydration stress (Hallsworth, 1998). Thus, we could hypothesize that 2-PE treatment induced a mutation in *HOG1* to create a hyperactive Hog1p.F138L enabling to better resist 2-PE toxicity by decreasing the available water loss *via* accumulation of glycerol, similar to the hyperosmotic stress response. However, whether or not glycerol accumulation contributes to 2-PE resistance is still an open question that will require additional work. Nonetheless, in support of a role of glycerol contributing to 2-PE resistance, it has been recently reported that high glycerol synthesis ability and the efficient HOG pathway may contribute to the high 2-PE tolerance of the stress-tolerant yeast *Candida glycerinogenes* WL2002-5 while converting 2-phenylalanine into 2-PE (Lu et al., 2016). The other effect of the hyperactivity of this Hog1p enzyme is likely to activate nuclear relocalization of the transcription factors Msn2p/Msn4p through its phosphorylation (Petrenko et al., 2013). Moreover, the expression of genes encoding these transcription factors were upregulated in the 2-PE-evolved strain, accounting for the strong upregulation of stress-related genes found in this strain, among which are genes implicated in reserve carbohydrates metabolism, as hallmarks of stress response (Parrou et al., 1999; Enjalbert et al., 2004). Whether the increased trehalose accumulation contributes to the resistance of strain C9 to 2-PE is a matter of debate, because on the one hand, it is well established that cells with high intracellular trehalose show better resistance to different stresses and higher viability (François and Parrou, 2001; Elbein et al., 2003; Kitichantaropas et al., 2016). On the other hand, the increase in trehalose production in the evolved strain could be a consequential event of the acquisition of the mutation in the *HOG1* gene that results in the upregulation of trehalose synthesis genes, *TPS1*, *TSL1* and *TPS2* through the activation of Msn2/4p.

The missense mutation in *PDE2* resulting in the variant Pde2p.P132S could additionally contribute to the stressful state of the C9 strain. Indeed, *PDE2* encodes a high-affinity cyclic AMP phosphodiesterase, whose overexpression can antagonize effects of hyperactive PKA pathway by reducing cAMP (Sass et al., 1986). As the cAMP-dependent PKA pathway is known to enhance sensitivity to several stress conditions (Sass et al., 1986; Casado et al., 2011; Molin et al., 2020), in part through the repression of Msn2p/Msn4p-dependent gene expression (Smith et al., 1998), it is therefore conceivable that the missense mutation in *PDE2* leads to a gain of function that increases the phosphodiesterase activity.

An alteration of cell wall structure was another significant physiological modification observed in the 2-PE-adapted C9 strain, as indicated by a greater resistance of this strain to the hydrolytic action of lyticase, an enzyme cocktail with β-1,3-glucan hydrolase and β-1,3-glucanase activity. This cell wall alteration could result from the conjugation of two mechanisms. The first one is upregulation of many cell wall-related genes that are typically involved in the cell wall compensatory mechanism triggered in response to cell wall damages (Lagorce et al., 2003; García et al., 2004), suggesting a cell wall compensatory mechanism in response to 2-PE treatment. This effect is likely a result of the hyperactive Hog1p.F138L variant resulting from *HOG1* mutation and the attendant activation of Msn2/4p transcription factor, since many cell wall genes have been reported to be upregulated through these factors (Berry and Gasch, 2008; Garcia et al., 2009). The other mechanism could be linked to the mutation in *CRH1* in C9 strain. This gene encodes one of the two chitin transglycosylases that are responsible for chitin to β-1,6-glucan cross-linkages (Rodriguez-Pena et al., 2000; Cabib, 2009). The missense mutation found in *CRH1* could have generated higher trans-glycosidase activity of Crh1p. These two mechanisms could be responsible for modification of the linkages between cell wall components, leading to higher resistance to the hydrolytic action of lyticase. In addition, mutation in *BNI1* and *SPA2* in the 2-PE-adapted C9 strain could also contribute to cell wall remodeling or cell wall repair at the bud site during growth since these two genes encode components of the polarisome that is needed for this polarized (unidimensional) cell growth (Pruyne and Bretscher, 2000). It was indeed shown that in response to cell wall damages caused by mechanical stress, there is a rapid depolarization at the growth sites, which involves disruption of actin cables and destabilization of the polarisome, resulting in growth inhibition. These events are then accompanied by uniform synthesis and strengthening of the stretched cell wall, thereby preventing potential cell lysis (Mishra et al., 2022). Interestingly, it was reported that this cell survival in response to a compressive mechanical stress was under the cooperative control of Pkc1p and calcineurin pathways, which are two major signaling pathways implicated in the cell wall compensatory response. Therefore, a similar process induced by 2-PE and probably mediated by Hog1-dependent osmotic stress pathway could also take place and account for growth reduction of the 2-PE-evolved strain, as compared to the reference strain.

Other mutations were found in C9 strain that could contribute to 2-PE resistance. This can be the case for the missense mutation of *FLR1.* This gene codes for a plasma membrane transporter of the major facilitator superfamily, a member of the drug H^+^-antiporter family (Goffeau et al., 1997). The missense mutation in this gene could result in a hyperactive Flr1p.P161S variant that is implicated in the expulsion of 2-PE out of the cell. Likewise, the mutation in *MAK5* gene (Mak5p.A215T) that encodes an essential nucleolar protein involved in large ribosome subunit biogenesis (Zagulski et al., 2003) may account for the reduced expression of genes that encode ribosomal proteins, which favors a stressful-state.

The C9 strain yet harbored many other mutations and transcriptional changes that at first glance do not seem to be related to the resistance to 2-PE. In particular, the missense mutation in *GAL80*, which encodes a transcriptional repressor of *GAL* genes (Sellick et al., 2008) was accompanied by the upregulation of several of the *GAL* genes, i.e., *GAL2*, *GAL10*, *GAL4* and *PGM2,* indicating that the Gal80p.H376Y variant encoded by the mutated *GAL80* has lost its repression activity. Likewise, strong upregulation of *MAL* genes is likely a consequence of hyperactive Hog1p that activated Msn2/4p-dependent genes, among which are most of the *MAL* genes. It is noteworthy that the upregulation of maltose and galactose genes, which are otherwise repressed by glucose in reference to yeast (Federoff et al., 1983), could enable C9 to utilize these alternative carbon sources together with glucose, although we have not examined this possibility.

It is known that when yeast cells gain resistance against a particular stress type, they may be either protected or be more susceptible to other stress types (Lewis et al., 1995; Hohmann, 2002). Thus, we tested the evolved strain C9 and the reference strain for their potential cross-resistance against a variety of stress types. Among various stressors tested, the evolved strain was slightly more sensitive to boron and cobalt stress than the reference strain, which can be explained by the well-known ‘trade-off’ situation in evolutionary engineering (Çakar et al., 2012), where the evolved strains may lose some properties while gaining new ones. In this work, the higher boron sensitivity of the evolved strain may be associated with the downregulation of *ATR1* (Supplementary Table S4) which encodes the main boron exporter in yeast. It was previously shown that *S. cerevisiae* cells overexpressing *ATR1* were boron-resistant (Kaya et al., 2009). Also, the higher sensitivity of C9 to iron may be associated with the downregulation of the genes related to iron metabolism (Supplementary Table S4), as these genes are involved in general iron uptake under iron depletion or cobalt stress (Stadler and Schweyen, 2002). In addition, the genes related to iron metabolism were upregulated in a cobalt-resistant *S. cerevisiae* strain (Çakar et al., 2009; Alkim et al., 2013). Overall, the increased sensitivity of C9 to these stressors is very likely a consequence associated with the physiological state of the strain adapted to 2-PE, but is neither involved in 2-PE-resistance nor in the metabolism of this molecule.

On the other hand, the finding of a higher resistance of the evolved strain against 2-phenylacetate brought out the idea that an easy way to make the cells resistant to 2-PE is to convert it to phenylacetate, which involves two consecutive oxidation reactions. Quite interestingly, our transcriptomic analysis unraveled a huge upregulation of *ALD3* (>200 fold) and *ALD4* (> 20 fold), which encode a cytoplasmic and a mitochondrial NAD^+^-aldehyde dehydrogenase, respectively. These enzymes can catalyze the oxidation of phenylacetaldehyde into phenylacetate, which has been already shown for Ald3p (Kim et al., 2014). The oxidation of 2-PE into phenylacetaldehyde could be also catalyzed by Ald3p/Ald4p or possibly by the oxidoreductase encoded by *BDH2* since this gene is homologous to *BDH1* which codes for an oxidoreductase that oxidizes 2,3-butanediol into acetoin (González et al., 2000), and the expression of *BDH2* is 21-fold increase in the 2-PE-adapted strain. Taken together, our data suggest that beyond inducing a stress state that likely contributes to 2-PE resistance, the upregulation of oxidoreductase and dehydrogenase genes activates a mechanism that detoxifies the yeast from 2-PE by transforming it into a less toxic molecule, 2-phenylacetate. It should be noted that activation of a detoxification mechanism is quite commonly found following the ALE strategy, as was obtained for furfural (Jung et al., 2017; Zheng et al., 2022) or coniferyl aldehyde resistance (Hacısalihoğlu et al., 2019). Our data also indicate that this detoxification mechanism must be suppressed in the engineering of highly 2-PE-producing yeast, consistent with the fact that deletion of *ALD3* combined with overexpression of *ARO9* and *ARO10* encoding a transaminase and decarboxylase, respectively, promoted high production of 2-PE from phenylalanine (Kim et al., 2014). These results also tell us that engineering a strain for high 2-PE production that must be at the same time highly resistant to this flavor could be achieved by adapting a strain defective in this detoxification mechanism.

## 5. Conclusion

In this work, we applied *in vivo* evolutionary engineering to adapt the yeast *S. cerevisiae* to the toxic compound 2-phenylethanol (2-PE). This ALE strategy led to the isolation of an adapted strain able to sustain growth in the presence of 3.4 g/L of 2-PE, which is roughly three times higher than the non-evolved strain. The genome sequencing of the evolved strain revealed numerous genes bearing single mutations, among which the most relevant was in the *HOG1* gene. The mutation in this gene resulted in a hyperactive Hog1p kinase which in turn activates the high osmolarity signaling pathway, as witnessed by up to 3 times higher production of glycerol in the evolved strain. In addition, our transcriptomic analysis implies that the resistance phenotype may be related to the Msn2/Msn4p-dependent stress response mediated by *HOG1* pathway, which also included activation of many cell wall-related genes, with a measurable consequence that the evolved strain being more resistant to lyticase, a cell wall-degrading cocktail containing endo β-1,3 glucanase and exo-β-1,3-glucanase activity. Furthermore, the finding of a huge upregulation of *ALD3* and *ALD4* encoding NAD^+^ aldehyde dehydrogenase, and *BDH2* encoding a putative oxidoreductase along with the finding that phenylacetate is much less toxic than 2-PE suggested the activation of a detoxification mechanism of 2-PE through its conversion into phenylacetate mediated by these dehydrogenases. Taken together, the 2-PE tolerance is a multigenic and complex trait that needs to be investigated further in light of the present study’s findings.

## Data availability statement

The datasets presented in this study can be found in online repositories. The names of the repository/repositories and accession number(s) can be found in the article/Supplementary material.

## Author contributions

ZÇ, GB, and JF: conceptualization, supervision, and funding acquisition. CH, BT-Y, ÜY, CA, MA, AT, and HK: methodology. CH, BT-Y, CA, MA, and AT: data curation and writing – original draft preparation. CH, AT, GB, JF, and ZÇ: writing, review and editing and project administration. All authors contributed to the article and approved the submitted version.

## Funding

This study was supported by Istanbul Technical University (ITU) Research Funds (BAP Project no: 36128, PI: ZÇ) and COST Action FA0907 (to GB and JF).

## Conflict of interest

The authors declare that the research was conducted in the absence of any commercial or financial relationships that could be construed as a potential conflict of interest.

## Footnotes

^1^ http://mips.helmholtz-muenchen.de/funcatDB

^2^ http://growthrate.princeton.edu/transcriptome/analyze.shtml

^3^ http://www.ncbi.nlm.nih.gov/geo/query/acc.cgi?acc=GSE59353

^4^ http://funspec.med.utoronto.ca/

^5^ http://www.yeastract.com/
