# Supplementary material for: Genomic, transcriptomic, and metabolic characterization of 2-Phenylethanol-resistant Saccharomyces cerevisiae obtained by evolutionary engineering
Source: Front Microbiol. 2023 Apr 11;14:1148065. doi: 10.3389/fmicb.2023.1148065 (PMC10127108; doi:10.3389/fmicb.2023.1148065)
Supplement: Supplementary file 1 [file Data_Sheet_1.pdf]

Supplementary Material

Genomic, Transcriptomic and Metabolic Characterization of 2-Phenylethanol-resistant *Saccharomyces cerevisiae* Obtained by Evolutionary Engineering

Can Holyavkin, Burcu Turanlı-Yıldız, Ülkü Yılmaz, Ceren Alkım, Mevlüt Arslan, Alican Topaloğlu, H. İbrahim Kısakesen, Gustavo de Billerbeck, Jean Marie François*, Z. Petek Çakar*

*** Correspondence:** Prof. Z. Petek Çakar: cakarp@itu.edu.tr , Prof. Jean-Marie François fran_jm@insa-toulouse.fr

1 Supplementary Data

Supplementary Material should be uploaded separately on submission. Please include any supplementary data, figures and/or tables.

Supplementary material is not typeset so please ensure that all information is clearly presented, the appropriate caption is included in the file and not in the manuscript, and that the style conforms to the rest of the article.

2 Supplementary Figures and Tables

For more information on Supplementary Material and for details on the different file types accepted, please see here.

2.1 Supplementary Figures

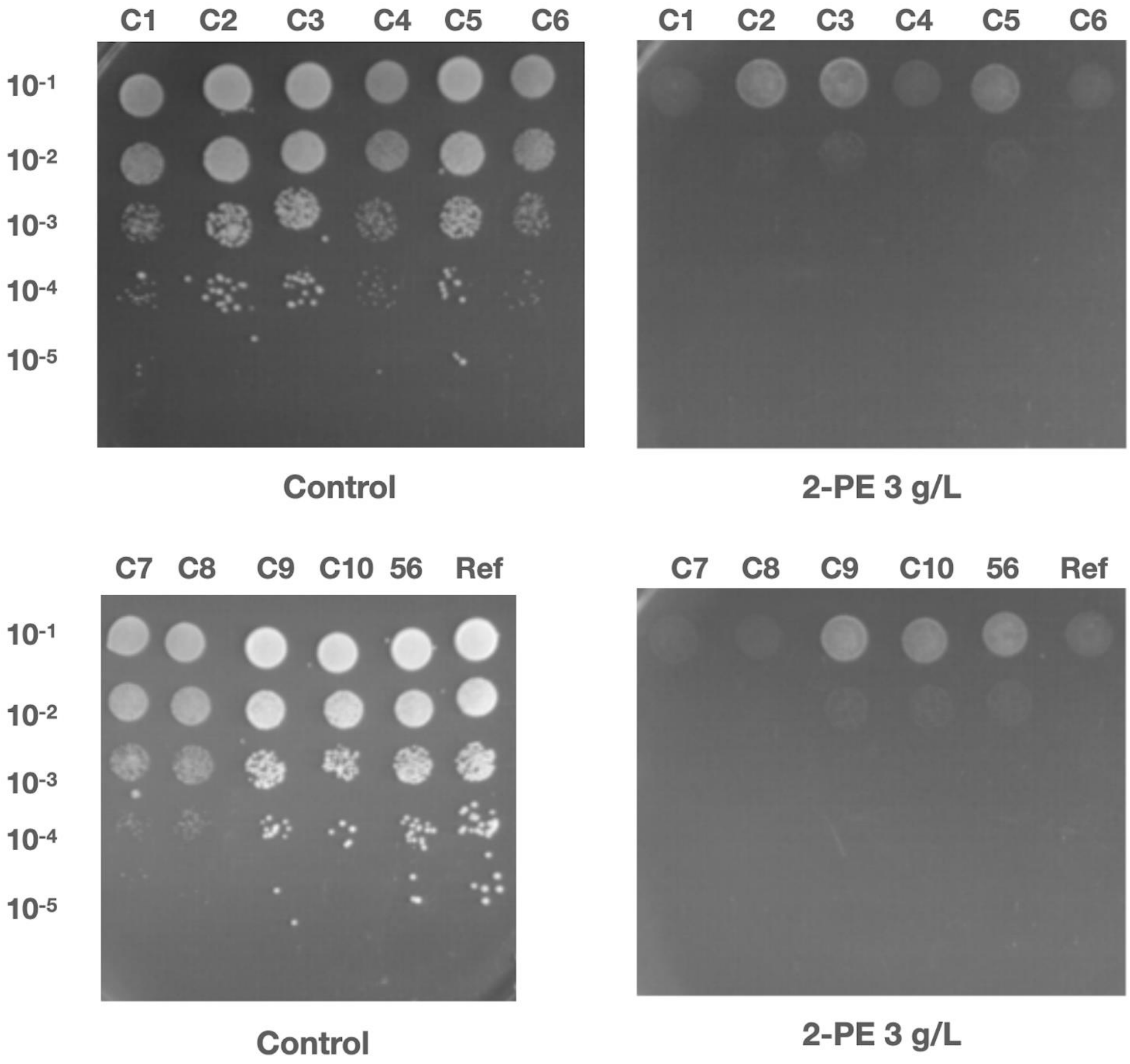

Supplementary Figure S1. 2-Phenylethanol-resistance spot assay results of the reference strain (Ref), the final (56th) population of selection (56) and the evolved strains C1- C10 under control, 2 g/L and 3 g/L 2-PE stress conditions.

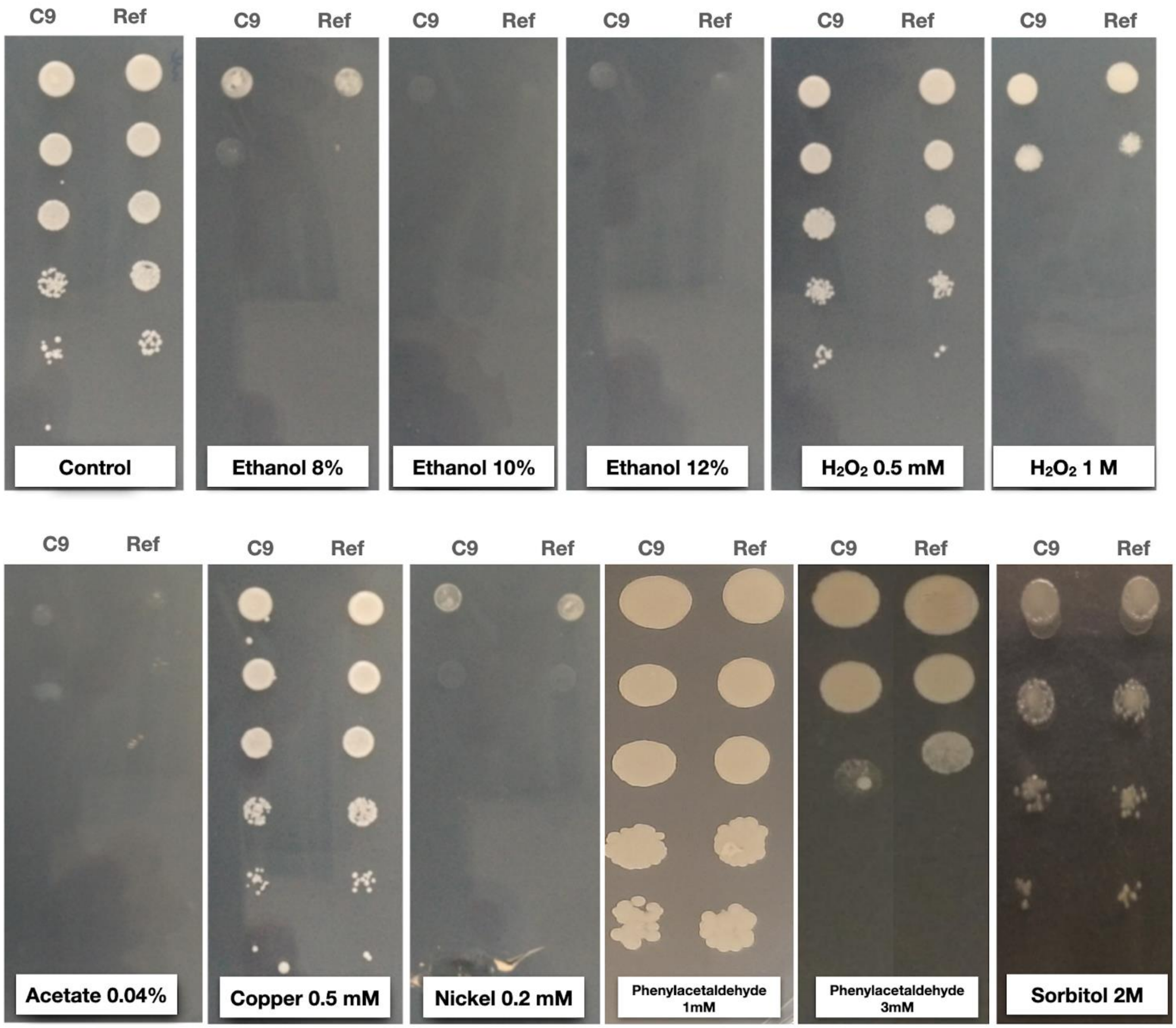

Supplementary Figure S2. Cross-resistance test results of the 2-phenylethanol-resistant evolved strain C9 and the reference strain (Ref) upon ethanol (8-10 and 12 % v/v), H_2O_2 (0.5 and 1.0 M), acetate (0.04%), copper (0.5 mM), nickel 0.2 mM, phenylacetaldehyde (1 mM and 3 mM) and sorbitol (2 M) stress.

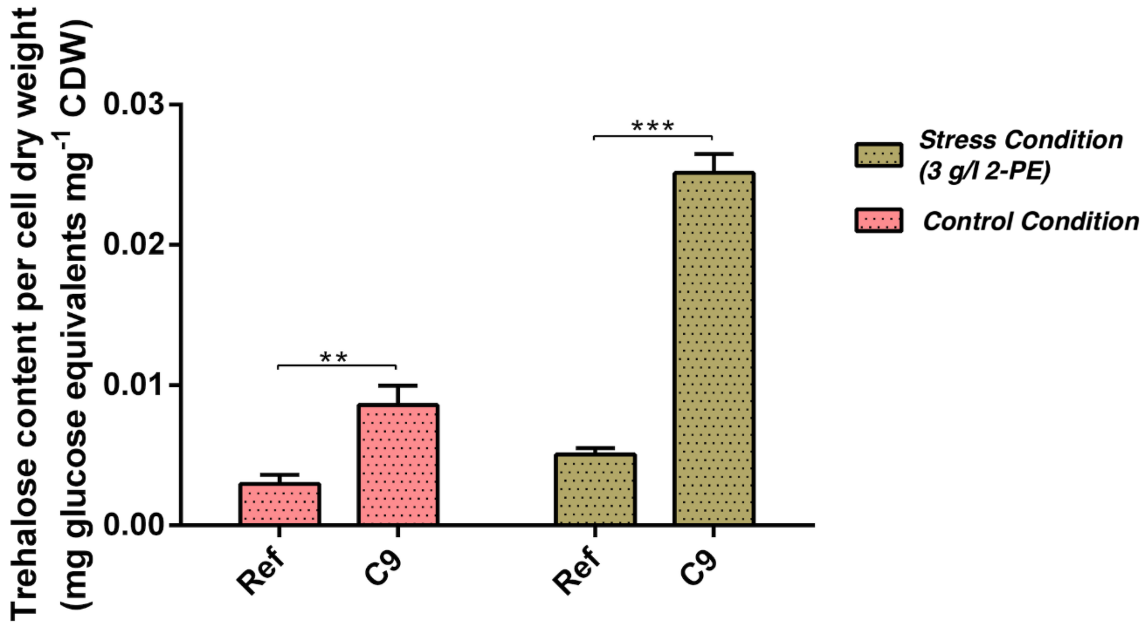

Supplementary Figure S3. Trehalose contents of C9 and the reference strain under control and 3 g/L 2-phenylethanol stress conditions.

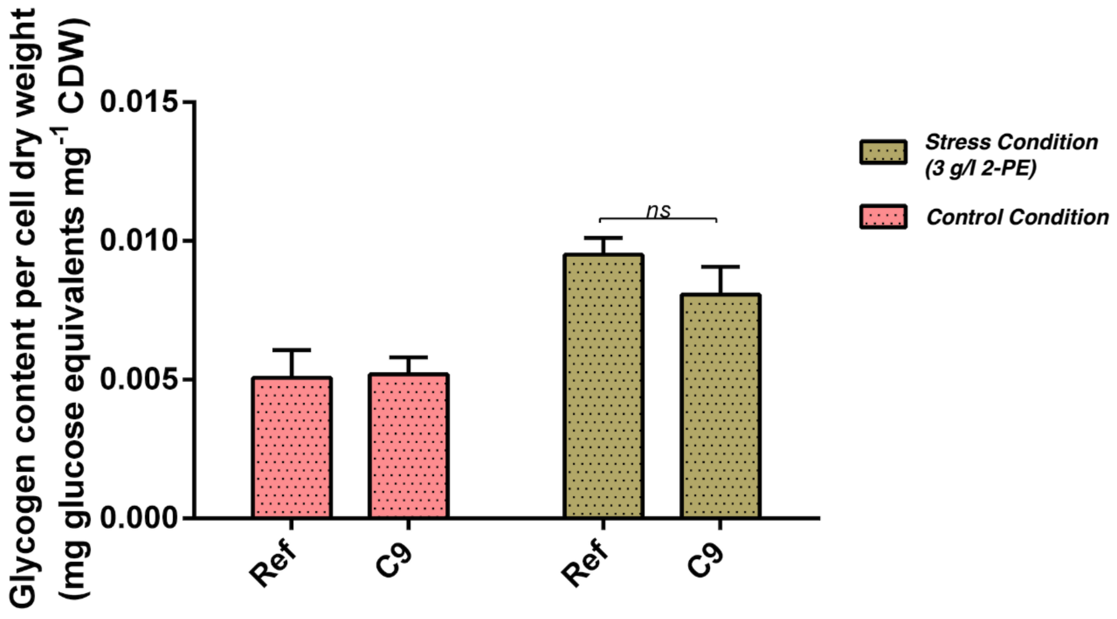

Supplementary Figure S4. Glycogen contents of C9 and the reference strain under control and 3 g/L 2-phenylethanol stress conditions.

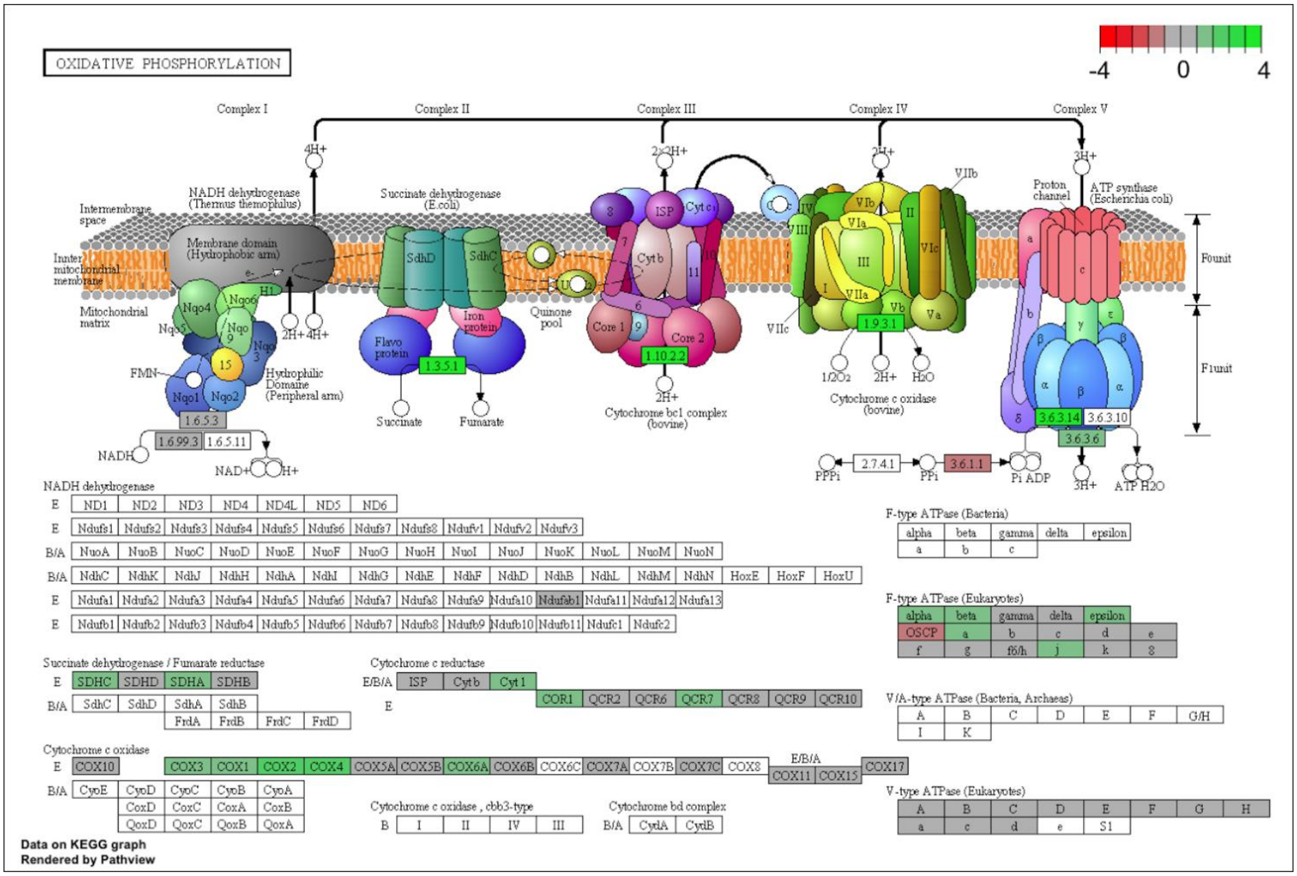

Suppl. Figure S5. KEGG pathway of the Oxidative Phosphorylation Pathway (KEGG ID sce00190) that belongs to the 2-phenylethanol-resistant strain C9. The green boxes indicate the upregulated genes, while the red ones indicate the downregulated genes.

2.2 Supplementary Tables

Supplementary Table S2. Final OD_{600} values of YMM cultures of the reference strain and the EMS-mutagenized initial population derived from the reference strain, both grown for 24 h at 30ºC under control and 2-phenylethanol stress (0.5-3 g/l) conditions.

Phenylethanol concentration (g/l)	Final OD_{600} of the Reference Strain	Final OD_{600} of the EMS-mutagenized Initial Population
0	5.84±0.29	5.92±0.31
0.5	5.69±0.26	5.48±0.29
1.0	5.65±0.27	5.77±0.28
1.5	5.24±0.25	5.23±0.26
2.0	4.74±0.23	4.98±0.25
2.5	4.02±0.22	4.04±0.25
3.0	2.54±0.13	2.74±0.16

Supplementary Table S2. The significantly (adjusted p value < 0.05) up- and downregulated genes related to the ATP synthase, ATP production and Oxidative Phosphorylation Pathway (KEGG ID: sce00190) in C9, in comparison with the reference strain, based on DNA microarray analysis.

ORF name	Gene name	Fold change	Function (Cherry et al., 2012)	Main TF involved*
YDL130W-A	*STF1*	3.271-fold increase	Protein involved in regulation of the mitochondrial F1F0-ATP synthase	Fhl1p, Msn4p
YJL116C	*NCA3*	2.002-fold increase	Protein involved in mitochondrion organization	Fhl1p, Msn4p, Mbp1p, Cbf1p, Cst6p, Ino4p, Mcm1p, Msn2p, Rap1p, Skn7p, Yap1p, Stb5p, Azf1p
Q0250	*COX2*	1.694-fold increase	Subunit II of cytochrome c oxidase (Complex IV)	
Q0045	*COX1*	1.276-fold increase	Subunit I of cytochrome c oxidase (Complex IV)	
YLR093C	*NYV1*	1.198-fold increase	v-SNARE component of vacuolar SNARE complex	**Msn2p**
Q0275	*COX3*	1.19-fold increase	Subunit III of cytochrome c oxidase (Complex IV)	
YML081C-A	*ATP18*	1.153-fold increase	Subunit j of the mitochondrial F1F0 ATP synthase	Hap4p, Stb5p, Hap2p, Rds2p, Abf1p
YOR065W	*CYT1*	1.06-fold increase	Cytochrome c1	Hap4p, Hap1p, Hap2p, Ste12p, Cbf1p, Rap1p, Sfp1p, Yrr1p
YBL045C	*COR1*	1.015-fold increase	Core subunit of the ubiquinol-cytochrome c reductase complex	Gcn4p, Hap4p, Cbf1p, Msn2p, Cst6p, Yap1p, Sok2p, Sut1p, Gis1p
YDR529C	*QCR7*	0.985-fold increase	Subunit 7 of ubiquinol cytochrome-c reductase (Complex III)	Hap4p
YBL099W	*ATP1*	0.857-fold increase	Alpha subunit of the F1 sector of mitochondrial F1F0 ATP synthase	Hap4p, Skn7p, Hap2p
YPL271W	*ATP15*	0.84-fold increase	Epsilon subunit of the F1 sector of mitochondrial F1F0 ATP synthase	Hap4p, Hap2p
YDR322C-A	*TIM11*	0.773-fold increase	Subunit e of mitochondrial F1F0-ATPase	Cin5p

YDR377W	*ATP17*	0.706-fold increase	Subunit f of the F0 sector of mitochondrial F1F0 ATP synthase	Hap4p, Aro80p, Rph1p
YLR393W	*ATP10*	0.474-fold increase	Assembly factor for the F0 sector of mitochondrial F1F0 ATP synthase	Ume6p
Q0130	*OLI1*	0.43-fold increase	F0-ATP synthase subunit c (ATPase-associated proteolipid)	
YBL102W	*SFT2*	0.362-fold increase	Tetra-spanning membrane protein found mostly in the late Golgi	Gcn4p
YPR020W	*ATP20*	0.355-fold increase	Subunit g of the mitochondrial F1F0 ATP synthase	Hap4p, Hap2p
YDL004W	*ATP16*	0.318-fold increase	Delta subunit of the central stalk of mitochondrial F1F0 ATP synthase	Cin5p, Hap4p, Mbp1p
YPL078C	*ATP4*	0.215-fold increase	Subunit b of the stator stalk of mitochondrial F1F0 ATP synthase	
YIL098C	*FMC1*	0.221-fold decrease	Mitochondrial matrix protein	Nrg1p
YIR024C	*INA22*	0.245-fold decrease	F1F0 ATP synthase peripheral stalk assembly factor	
YKL016C	*ATP7*	0.25-fold decrease	Subunit d of the stator stalk of mitochondrial F1F0 ATP synthase	Msn4p, Hap4p, Cbf1p, Rap1p, Gcn4p, Rpn4p
YPL099C	*INA17*	0.426-fold decrease	F1F0 ATPase synthase peripheral stalk assembly factor	
YMR064W	*AEP1*	0.429-fold decrease	Protein required for expression of the mitochondrial OLI1 gene	Gcn4p
YDR350C	*ATP22*	0.46-fold decrease	Specific translational activator for the mitochondrial ATP6 mRNA	
YDL181W	*INH1*	0.624-fold decrease	Protein that inhibits ATP hydrolysis by the F1F0-ATP synthase	
YDR298C	*ATP5*	0.834-fold decrease	Subunit 5 of the stator stalk of mitochondrial F1F0 ATP synthase	Hap4p

YNR020C	*ATP23*	1.109-fold decrease	Putative metalloprotease of the mitochondrial inner membrane	Abf1p
YNL315C	*ATP11*	1.195-fold decrease	Molecular chaperone	Fhl1p, Msn4p

*The analysis for main transcription factors (TF) was performed using YEASTRACT database and Search for TFs tool with the following settings:

- TF acting as activator
- DNA binding and expression evidence.

Supplementary Table S3. The upregulated genes with more than 10-fold change in C9, in comparison with the reference strain, based on DNA microarray analysis.

ORF name	Gene name	Fold change	Function (Cherry et al., 2012)	Main TF involved*
		**Carbohydrate metabolism**		
YGR292W	*MAL12*	207.4	Maltase (alpha-D-glucosidase)	**Msn2p**, Swi4p
YBR299W	*MAL32*	198.7	Maltase (alpha-D-glucosidase)	**Msn2p**, Msn4p, Ste12p
YGR289C	*MAL11*	127.9	High-affinity maltose transporter	Cin5p, **Msn2p**
YBR298C	*MAL31*	18.0	Maltose permease, high-affinity maltose transporter	**Msn2p**, Msn4p
YCL040W	*GLK1*	10.8	Glucokinase	Fhl1p, **Msn2p**, Msn4p, Gcn4p, Gcr1p, Gcr2p, Rap1p, Ino2p, Tye7p
YDR516C	*EMI2*	18.4	Nonessential protein of unknown function, expression regulated by glucose-repression transcription factors Mig1/2p	Fhl1p, **Msn2p**, Pdr1p
YFR053C	*HXK1*	223.6	Hexokinase isoenzyme 1	**Msn2p**, Msn4p, Gcr1p, Ino2p, Skn7p, Tec1p
YDR343C	*HXT6*	19.9	High-affinity glucose transporter	**Msn2p**, Msn4p, Gcn4p, Ino2p, Adr1p, Rim101p
YDR342C	*HXT7*	26.3	High-affinity glucose transporter	**Msn2p**, Gcn4p, Ino2p, Pdr1p, Yrr1p, Ino4p
YDL021W	*GPM2*	13.9	Homolog of Gpm1p phosphoglycerate mutase	Cin5p, Gcn4p, Rap1p, Sok2p
YGL156W	*AMS1*	15.6	Vacuolar alpha mannosidase, involved in free oligosaccharide degradation	
YMR105C	*PGM2*	100.2	Phosphoglucomutase	
**Glycogen and trehalose biosynthesis**				**Main TF involved**
YEL011W	*GLC3*	28.1	Glycogen branching enzyme, involved in glycogen accumulation	**Msn2p**

YFR015C	*GSY1*	20.7	Glycogen synthase	**Msn2p**, Ixr1p
YLR258W	*GSY2*	17.7	Glycogen synthase	**Msn2p**, Yap1p, Ino2p
YPR160W	*GPH1*	217.1	Glycogen phosphorylase required for glycogen mobilization	Cin5p, Hsf1p, **Msn2p**, Yrr1p
YOR178C	*GAC1*	36.8	Regulatory subunit for Glc7p type-1 protein phosphatase (PP1)	Cin5p, Fhl1p, **Msn2p**, Ste12p, Gcn4p, Tye7p, Tec1p
YDR074W	*TPS2*	12.8	Phosphatase subunit of the trehalose-6-P synthase/phosphatase complex	**Msn2p**, Msn4p, Gcn4p, Crz1p, Hot1p, Skn7p
YML100W	*TSL1*	67.3	Large subunit of trehalose-6-phosphate synthase/phosphatase complex	Hsf1p, **Msn2p**, Msn4p, Crz1p, Swi4p, Rlm1p, Met32p
YKL035W	*UGP1*	12.2	UDP-glucose pyrophosphorylase	Fhl1p, Hsf1p, **Msn2p**, Crz1p, Skn7p, Sko1p
**Pentose phosphate shunt**				
YBR117C	*TKL2*	208.6	Transketolase, required for aromatic amino acid synthesis	Cin5p, Fhl1p, Hsf1p, **Msn2p**, Yap1p, Zap1p
YGR248W	*SOL4*	26.0	6-phosphogluconolactonase	**Msn2p**, Yap1p, Ste12p, Rap1p, Swi4p, Mga1p
YGR043C	*NQM1*	17.8	Transaldolase of unknown function	Cin5p, **Msn2p**, Ste12p, Gcn4p, Rap1p, Hot1p, Skn7p, Sko1p, Stb5p
**Other metabolic processes**				
YMR169C	*ALD3*	234.0	Cytoplasmic aldehyde dehydrogenase, involved in beta-alanine synthesis	**Msn2p**, Sko1p
YOR374W	*ALD4*	28.1	Mitochondrial aldehyde dehydrogenase, required for growth on ethanol and conversion of acetaldehyde to acetate	**Msn2p**, Msn4p, Gcn4p, Gcr1p, Crz1p, Adr1p, Stb5p
YAL061W	*BDH2*	21.1	Putative medium-chain alcohol dehydrogenase	Fhl1p, **Msn2p**, Ste12p, Crz1p, Pdr1p, Bas1p

**Response to stress**				
YOR049C	*RSB1*	10.9	Suppressor of sphingoid long chain base (LCB) sensitivity of an LCB-lyase mutation	Oaf1p
YDL222C	*FMP45*	101.7	Integral membrane protein localized to mitochondria, required for sporulation and maintaining sphingolipid content	Fhl1p, **Msn2p**, Msn4p, Gcn4p
YDR258C	*HSP78*	10.4	Oligomeric mitochondrial matrix chaperone, involved in mitochondrial thermotolerance after heat shock	Hsf1p, **Msn2p**, Yap1p, Msn4p, Gcn4p, Pdr1p, Yrr1p
YER103W	*SSA4*	16.2	Heat shock protein, involved in SRP-dependent cotranslational protein-membrane targeting and translocation	Cin5p, Fhl1p, Hsf1p, **Msn2p**, Yap1p, Msn4p, Ste12p, Yrr1p, Ecm22p
YGR088W	*CTT1*	52.5	Cytosolic catalase T, plays a role in protection from oxidative damage by hydrogen peroxide	**Msn2p**, Msn4p, Ste12p, Gcn4p, Skn7p, Hap1p
YGR243W	*FMP43*	14.2	Highly conserved subunit of mitochondrial pyruvate carrier	
YIL101C	*XBP1*	19.8	Transcriptional repressor, binds to promoter sequences of the cyclin genes *CYS3* and *SMF2*	Cin5p, **Msn2p**, Msn4p, Gcn4p, Crz1p, Skn7p, Swi4p, Rox1p
YOL052C-A	*DDR2*	58.9	Multi-stress response protein	Cin5p, **Msn2p**, Yap1p, Msn4p, Ste12p, Gcn4p, Sok2p, Skn7p, Gis1p, Leu3p, Mig1p, Sfl1p, Met32p, Xbp1p
YMR173W	*DDR48*	10.1	DNA damage-responsive protein	**Msn2p**, Yap1p, Msn4p, Ste12p, Gcn4p, Sok2p, Skn7p, Tec1p
**Response to oxidative stress**				
YFL014W	*HSP12*	136.6	Plasma membrane protein involved in maintaining membrane organization	Hsf1p, **Msn2p**, Yap1p, Msn4p, Rap1p, Sok2p, Hot1p, Skn7p, Yrr1p, Adr1p, Cst6p, Gis1p, Leu3p, Mig1p, Msn1p, Sko1p, Swi4p, Tec1p, Yap5p, Yox1p, Sfl1p, Rox1p, Gzf3p

YMR250W	*GAD1*	19.4	Glutamate decarboxylase, converts glutamate into gamma-aminobutyric acid (GABA)	Gcn4p, Hcm1p
YHR104W	*GRE3*	11.9	Aldose reductase	Hsf1p, **Msn2p**, Msn4p
YOR120W	*GCY1*	11.0	Glycerol dehydrogenase	Cin5p, **Msn2p**, Gcn4p, Stb5p, Reb1p, Gal4p

*The analysis for main transcription factors (TF) was performed using YEASTRACT database and Search for TFs tool with the following settings:

- TF acting as activator
- DNA binding and expression evidence.

Supplementary Table S4. The downregulated genes with more than 4-fold change in C9, in comparison with the reference strain, based on DNA microarray analysis.

ORF name	Gene name	Fold change	Function (Cherry et al., 2012)	Main TF involved*
**Ribosome biogenesis**				
YMR131C	*RRB1*	4.9	Nuclear protein involved in early steps of ribosome biogenesis	Cbf1p
YOL010W	*RCL1*	5.6	Endonuclease that cleaves pre-RNA at site A2 for 18S rRNA biogenesis	
YNL075W	*IMP4*	5.4	Component of the SSU processome which is required for pre-18S rRNA processing	Gcn4p
YNL112W	*DBP2*	9.4	ATP-dependent RNA helicase of the DEAD-box protein family, involved in rRNA processing	**Msn2p**, Msn4p, Abf1p, Yrr1p, Put3p
YNR054C	*ESF2*	5.1	Essential nucleolar protein involved in pre-18S rRNA processing	
YOR145C	*PNO1*	8.5	Essential nucleolar protein involved in pre-18S rRNA processing	**Msn2p**
**Ribosomal subunit biogenesis**				
YAL025C	*MAK16*	5.9	Essential nuclear protein, constituent of 66S pre-ribosomal particles.	
YDR101C	*ARX1*	4.8	Nuclear export factor for the ribosomal pre-60S subunit	
YLR009W	*RLP24*	5.8	Essential protein required for ribosomal large subunit biogenesis	
YDR496C	*PUF6*	4.8	Pumilio-homology domain protein, involved in ribosomal large subunit biogenesis	**Msn2p**, Msn4p, Yap1p

YHR052W	*CIC1*	6.8	Essential protein that interacts with proteasome components, involved in ribosomal large subunit biogenesis	Yap1p
**Ribosomal large subunit assembly**				
YCR072C	*RSA4*	7.0	WD-repeat protein involved in ribosome biogenesis	Gcn4p
YIR026C	*YVH1*	5.4	Protein phosphatase, involved in vegetative growth at low temperatures , sporulation and glycogen accumulation	Dal82p
YKL009W	*MRT4*	6.2	Protein involved in mRNA turnover and ribosome assembly	Met31p, Cup9p, Tye7p
YDR299W	*BFR2*	6.0	Essential protein that is a component of 90S preribosomes, may be involved in rRNA processing	Ino2p
YLR276C	*DBP9*	5.5	DEAD-box protein required for 27S rRNA processing	
YPL211W	*NIP7*	6.4	Nucleolar protein required for 60S ribosome subunit biogenesis	Gcn4p, Tye7p
YKR081C	*RPF2*	5.1	Essential protein involved in rRNA maturation and ribosomal assembly, involved in the processing of pre-rRNA and the assembly of the 60S ribosomal subunits.	**Msn2p**, Yap1p
**Ribosomal large subunit export from nucleus**				

YAL059W	*ECM1*	9.3	Pre-ribosomal factor involved in 60S ribosomal protein subunit export	Adr1p
YLL034C	*RIX7*	5.1	Putative ATPase of the AAA family, required for the export of pre-ribosomal large subunits from the nucleus	Met31p, Aft2p, Gln3p, Reb1p, Rim101p, Rph1p
YOR294W	*RRS1*	4.7	Essential protein that binds ribosomal protein L11	
YDR361C	*BCP1*	5.5	Essential protein involved in nuclear export of Mss4p, a lipid kinase that plays a role in actin cytoskeleton organization and vesicular transport	Yap1p
**rRNA processing**				
YKL172W	*EBP2*	5.1	Required for 25S rRNA maturation and 60S ribosomal subunit assembly	
YKL078W	*DHR2*	7.6	Predominantly nucleolar DEAH-box ATP-dependent RNA helicase, required for 18S rRNA synthesis	
YGR159C	*NSR1*	7.2	Nucleolar protein that binds nuclear localization sequences, required for pre-rRNA processing and ribosome biogenesis	Msn4p, Pdr1p, Yrr1p
YHR085W	*IPI1*	5.3	Component of the Rix1 complex, involved in rRNA processing	
YNL182C	*IPI3*	5.3	Component of the Rix1 complex, involved in rRNA processing	Abf1p
YHR072W-A	*NOP10*	4.7	Subunit of box H/ACA snoRNP complex, required for pseudouridylation and processing of pre-18S rRNA.	
**Transmembrane transport**				

YML123C	*PHO84*	16.8	High-affinity inorganic phosphate transporter	**Msn2p**, Gcn4p, Ino2p, Ste12p, Fhl1p, Met31p, Yrr1p, Bas1p, Rpn4p, Stp2p, Nrg1p, Met4p, Put3p
YDR384C	*ATO3*	7.4	Plasma membrane protein, putative ammonium transporter	**Msn2p**, Gcn4p, Oaf1p, Ste12p
YGL255W	*ZRT1*	18.6	High-affinity zinc transporter of the plasma membrane, responsible for the majority of zinc uptake	**Msn2p**, Gcn4p, Ino2p, Met31p, Mot3p, Spt23p, Stp1p, Sum1p
YPR194C	*OPT2*	8.2	Oligopeptide transporter, also involved in mature vacuole formation	Ecm22p
YOL163W		4.9	Putative protein of unknown function, involved in transmembrane transport	
YML116W	*ATR1*	5.3	Multidrug efflux pump of the major facilitator superfamily	Yap1p, Tup1p, Rim101p
YBR294W	*SUL1*	19.8	High-affinity sulfate permease of the SulP anion transporter family	**Msn2p**, Msn4p
YJR124C		5.3	Putative protein of unknown function, expression induced under calcium shortage	
YER056C	*FCY2*	7.5	Purine-cytosine permease, mediates purine and cytosine accumulation	**Msn2p**, Msn4p, Gcn4p, Ino2p, Abf1p, Cbf1p
YPL092W	*SSU1*	5.5	Plasma membrane sulfite pump involved in sulfite metabolism	Ndt80p
YLL061W	*MMP1*	6.8	High-affinity S-methylmethionine permease	Met31p
**Mitochondrial citrate transport**				
YBR291C	*CTP1*	5.5	Mitochondrial inner membrane citrate transporter	
YMR241W	*YHM2*	5.1	Citrate and oxoglutarate carrier protein	**Msn2p**, Gcn4p, Ste12p
**Iron metabolism**				

YER145C	*FTR1*	4.7	High-affinity iron permease	**Msn2p**, Fhl1p, Ndt80p, Sfp1p, Sok2p, Tec1p, Tup1p
YMR058W	*FET3*	10.9	Ferro-O2-oxidoreductase	**Msn2p**, Gcn4p, Ste12p, Cbf1p, Fhl1p, Aft2p
YOL158C	*ENB1*	4.9	Endosomal ferric enterobactin transporter	Gcn4p, Put3p

*The analysis for main transcription factors (TF) was performed using YEASTRACT database and Search for TFs tool with the following settings:

- TF acting as activator
- DNA binding and expression evidence.
